# Amodal Aspects of Linguistic Design

**DOI:** 10.1371/journal.pone.0060617

**Published:** 2013-04-03

**Authors:** Iris Berent, Amanda Dupuis, Diane Brentari

**Affiliations:** 1 Department of Psychology, Northeastern University, Boston, Massachusetts, United States of America; 2 Department of Linguistics, University of Chicago, Chicago, Illinois, United States of America; Northeastern University, United States of America

## Abstract

All spoken languages encode syllables and constrain their internal structure. But whether these restrictions concern the design of the language system, broadly, or speech, specifically, remains unknown. To address this question, here, we gauge the structure of signed syllables in American Sign Language (ASL). Like spoken languages, signed syllables must exhibit a single sonority/energy peak (i.e., movement). Four experiments examine whether this restriction is enforced by signers and nonsigners. We first show that Deaf ASL signers selectively apply sonority restrictions to syllables (but not morphemes) in novel ASL signs. We next examine whether this principle might further shape the representation of signed syllables by nonsigners. Absent any experience with ASL, nonsigners used movement to define syllable-like units. Moreover, the restriction on syllable structure constrained the capacity of nonsigners to learn from experience. Given brief practice that implicitly paired syllables with sonority peaks (i.e., movement)—a natural phonological constraint attested in every human language—nonsigners rapidly learned to selectively rely on movement to define syllables and they also learned to partly ignore it in the identification of morpheme-like units. Remarkably, nonsigners failed to learn an unnatural rule that defines syllables by handshape, suggesting they were unable to ignore movement in identifying syllables. These findings indicate that signed and spoken syllables are subject to a shared phonological restriction that constrains phonological learning in a new modality. These conclusions suggest the design of the phonological system is partly amodal.

## Introduction

All spoken languages construct words from meaningless elements [1]. The word *elbow*, for instance, comprises two syllables—abstract meaningless units, whose internal structure is systematically restricted. Indeed, English speakers, for instance, accept syllables like *blog*, but they disallow *lbog*. Such observations suggest people possess systematic knowledge concerning the patterning of meaningless linguistic elements. Their knowledge is called *phonology*.

Phonological restrictions have been documented in all spoken languages, and some of these principles are arguably universal [2], [3]. But whether this design concerns speech [4], [5], specifically, or language, broadly [6], remains an open empirical question. To address this issue, we turn to the structure of sign languages. We reason that, if human brains share broad restrictions on language structure, then the phonological systems of signed and spoken languages will converge on their design. Distinct languages might share phonological primitives and constraints that apply to both speech and sign. Consequently, people should be able to extend their phonological knowledge to a novel linguistic modality. In line with this possibility, here, we show that fluent ASL signers impose systematic restrictions on the structure of syllables in American Sign Language (ASL), and these restrictions mirror the ones found in spoken languages. We next demonstrate that similar biases guide the behavior of English speakers who have had no previous experience with a sign language, and they constrain their capacity to extract ASL syllables. These results suggest that the design of the phonological mind is partly amodal.

Our investigation specifically concerns the syllable and the restrictions on its internal structure. Syllables are universal primitives of phonological organization in all spoken languages. They explain, for instance, the above-mentioned ban on sequences like *lbog* and the admittance of the same *lb-*sequence in *elbow*. Specifically, in *elbow*, the critical *lb* cluster spans different syllables, whereas in *lbog*, it forms the onset of a single syllable. Syllable structure, in turn, is subject to sonority restrictions.

Sonority is a scalar phonological property [7], [8] that correlates with the loudness of segments [9]: louder segments such as vowels are more sonorous than quieter segments, such as stop consonants (e.g., *b, p*). All syllables must exhibit a single peak of sonority, preferably, a vowel. Words like *can* exhibit a single vowel, so they are monosyllabic; in *candy*, there are two sonority peaks (two vowels), so it is a disyllable. Sonority restrictions are specifically phonological, as they constrain the structure of the syllable (i.e., meaningless phonological constituents) irrespective of the number of morphemes—meaningful units. The word *cans* and *candies*, for instance, comprise one vs. two syllables, respectively, even though both forms are bimorphemic (a base and the plural suffix). The existence of words like *cans*, with two morphemes, but a single sonority peak, indicates that sonority selectively constrains syllable structure—it is not necessarily relevant to morphemes.

**Figure 1:**
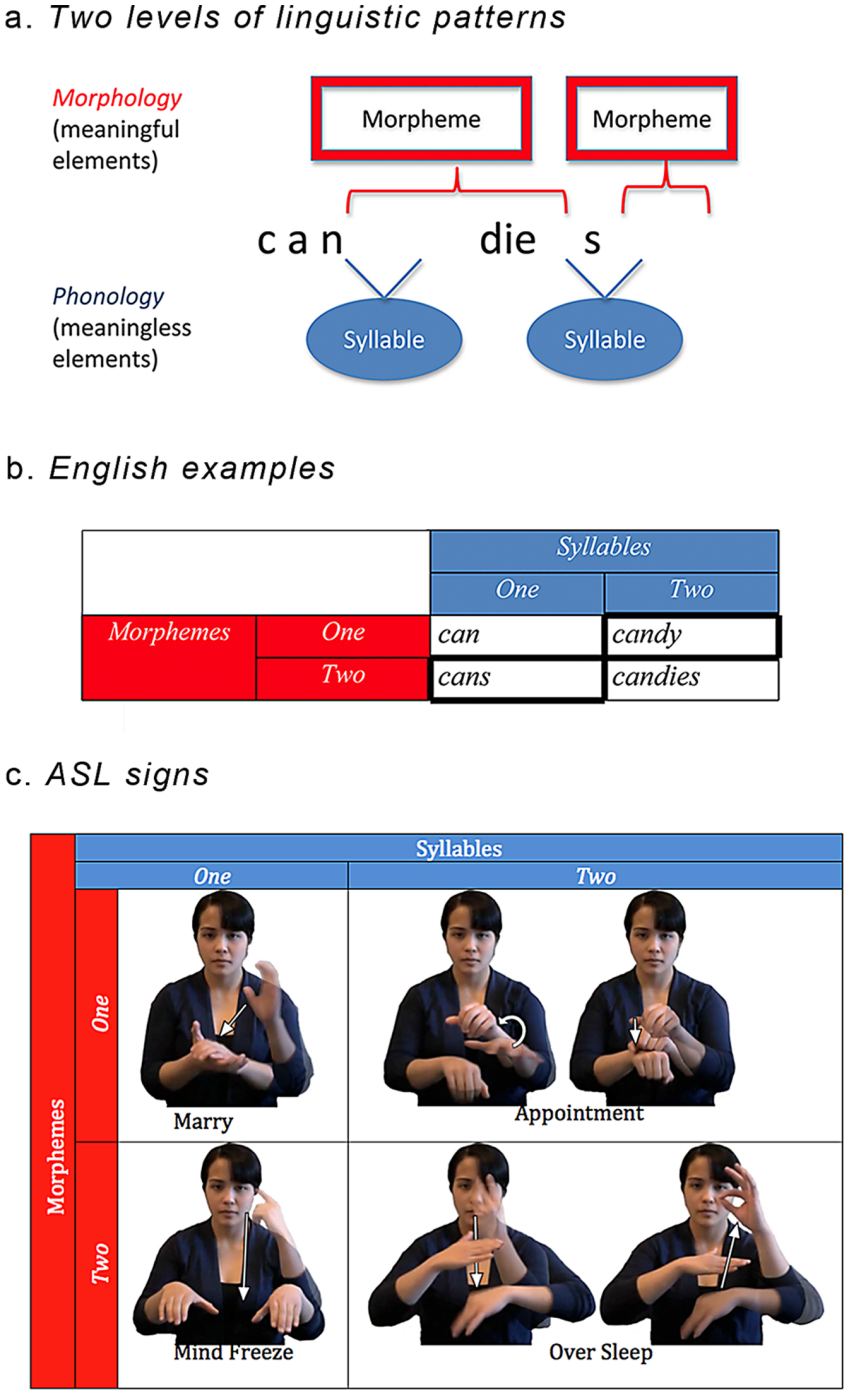
Syllables and morphemes across modalities. Panel *a* illustrates the pattern of meaningful elements (morphemes) and meaningless elements (syllables) in an English word. Panels *b-c* illustrate the manipulation of syllable and morpheme structure in English words (*b*) and ASL signs (*c*). Note that one-syllable signs have a single movement, whereas two-syllable signs have two movements (marked by arrows). Morphemes, by contrast, are defined by the number of handshapes. For example, the monomorphemic monosyllabic sign MARRY has a single group of active fingers (the open hand with the thumb extended) whereas in the monosyllabic bimorphemic sign MIND-FREEZE there are two groups of active fingers, the “one” (an extended index finger) handshape changes to an open hand with the thumb extended.

Linguistic analysis suggests that this phonological design might be shared across modalities. Like spoken language, signed languages comprise patterns of meaningless syllables and they require syllables to exhibit a single sonority peak [10]–[15]. But in sign languages, these sonority peaks typically correspond to movement—a peak of visual energy. Specifically, monosyllabic signs must include one movement, whereas disyllabic signs include two movements. Figure 1 illustrates this contrast for the ASL signs MARRY (a monosyllable with a single movement) and APPOINTMENT (a disyllabic sign with two movements). As in spoken languages, syllable structure in sign language is orthogonal to morphological organization. MIND-FREEZE, for instance, has a single movement, so it is monosyllabic, even though it comprises two morphemes, whereas APPOINTMENT is a disyllabic sign with two movements, but only one morpheme. Such observations underscore the selective application of sonority restrictions to syllables, not morphemes. This similarity in the organization of signed and spoken phonological systems suggests that the syllable might be an amodal phonological primitive, subject to a universal restriction on sonority. While specific linguistic proposals disagree on their detailed account of sonority in spoken [7], [16]–[19] and signed [14], [15], [20]–[26] languages, the broad requirement for a syllable to exhibit a single peak of sonority/energy is uncontroversial.

Past experimental work in spoken languages provides ample support for the representation of the syllable in both adults [27]–[32] and young infants [33]. Furthermore, there is evidence that people are sensitive to broad sonority restrictions, and they extend this knowledge even to syllable types that are unattested in their language [34]–[42]. For example, linguistic analysis[7], [8] suggests that syllables like *bnif* are preferred to *lbif*, as their sonority profile is better formed. Remarkably, similar preferences have been documented experimentally among speakers of various languages (English [34], [36]–[39], [43], [44], Spanish[40] and Korean[35]) despite no experience with either type of syllable. Such observations suggest that people encode broad phonological restrictions on the syllable structure of spoken language. However, less is known on the phonological organization of signs.

Previous research has shown that signers extract the phonological features of handshape, location and movement [45]–[50]. In fact, the capacity to encode handshape feature categorically is even present in four-month-old infants, irrespective of their exposure to sign [51], [52]. Signers are also sensitive to phonological well-formedness, as they are better able to detect a real sign embedded in a nonsense-sign context when the context is phonotactically licit [53]. These experimental results, however, do not establish whether the phonological representation of signs encodes syllable-structure, specifically. While signers can demonstrably identify syllable-like chunks in natural [54] and backward signing [55], and they can distinguish “one vs. two signs” in novel stimuli [56], past research did not dissociate the role of syllables from morphological constituents. Other findings, showing that signed syllables lack perceptual peaks [57] would seem to challenge the role of syllables altogether. Accordingly, there is currently no experimental evidence that signers effectively distinguish between syllables and morphemes. No prior experimental study has examined whether nonsigners can use sonority peaks to extract syllables from signs, and whether sonority principles constrain their ability to learn the structure of signed phonological systems. Our research examines these questions.

To determine whether signers and nonsigners are sensitive to syllable structure, we presented participants with short videos featuring novel ASL signs. These novel signs were organized in quartets that cross the number of syllables (either one or two syllables) with the number of morphemes (one or two morphemes). Syllable structure was defined by the number of movements—signs with one movement were considered monosyllabic; signs with two movements were defined as disyllabic.

**Figure 2:**
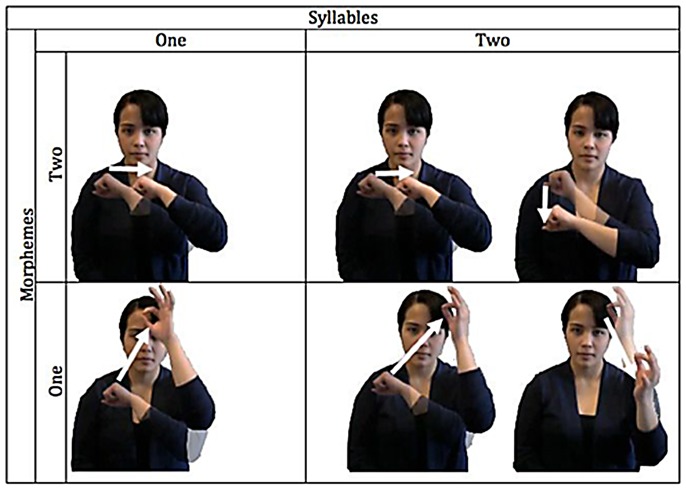
The distinction between syllables and morphemes in the novel ASL stimlus items. Note that one-syllable signs have a single movement, whereas two-syllable signs have two movements (marked by arrows). Morphemes, by contrast, are defined by the number of handshapes. For example, the monomorphemic monosyllabic sign has one group of active fingers (the closed fist with the thumb positioned infront of the fingers, the “S” handshape in ASL) whereas in the monosyllabic bimorphemic sign, there are two groups of active fingers - the “S” handshape changes to an “F” handshape (the tip of the pointer finger touching the tip of the thumb to form a small circle with the other three fingers extended).

We also manipulated the morphological structure of these novel signs. Although nonce words (signed or spoken) lack meaning, they can exhibit morphological structure. English speakers, for example, encode nonce words like *blixes* as bimorphemic, and subject them to grammatical restrictions that specifically appeal to morphological structure (e.g., the ban on regular plurals in compounds, *blixes-eater) [58]–[60]. Indeed, morphemes are abstract formal categories. While typical instances of a morpheme (e.g., *dog*, the noun-base of *dogs*) correspond to form-meaning pairings (e.g., *dog = */dog/-[CANINE]), morphemes are defined by formal restrictions. Phonological co-occurrence restrictions offer one criterion for the individuation of morphemes, and speakers demonstrably extend such restrictions to novel words [61]–[64]. We likewise used phonological restrictions to define the morphological structure of novel signs. Specifically, ASL requires a morpheme to exhibit a single group of active fingers (as well as location)[12], [13], [65]. Accordingly, signs with two groups of active fingers are invariably bimorphemic, whereas many signs with a single group are monomorphemic—this association between handshape and morphological structure is most clearly evident in the structure of ASL compounds [65]. An inspection of Figure 1 indeed shows that the compounds MIND-FREEZE and OVERSLEEP each exhibits a change in handshape, whereas the monomorphemic signs for MARRY and APPOINTMENT each exhibits a single handshape. Our experiments thus used handshape complexity to manipulate morphological structure. Signs with a single handshape were considered to be monomorphemic; those with two handshapes were bimorphemic. Within each morphological category, half of the items was monosyllabic (with one movement) whereas the other half was disyllabic (with two movements). As shown in Figure 2, monosyllabic and disyllabic signs were closely matched for their handshape, orientation, location and movement.

These materials were employed in two tasks. In the syllable count task, participants were asked to judge the number of syllables while ignoring the number of morphemes. The morpheme task, in turn, required participants to determine the number of morphemes while ignoring syllable structure. We provided participants with a brief explanation of the distinction between meaningless units (syllables) and meaningful ones (morphemes) and practice using both existing ASL signs and novel signs. However, participants received no explicit instruction on the principles that define signed syllables and morphemes. Experiment 1 presented these materials to a group of fluent ASL signers; Experiments 2–4 gauged their identification by English speakers who had no previous experience with a sign language.

If signers are sensitive to signed syllable structure, then syllable count should depend on sonority peaks, such that signs with one movement should be considered monosyllabic, and those with two movements should be disyllabic. It is conceivable, however, that signers might extract such units by relying on visual salience alone, rather than linguistic principles that specifically link sonority/energy peaks to syllables. The morpheme count task allows us to test this possibility. Unlike syllables, morphemes in our materials are defined by handshape, rather than by movement. If signers segment signs based on visual salience, then they should invariably rely on movement, irrespective of whether they count syllables or morphemes. If, however, they extract phonological or phonetic constituents that specifically link visual salience to syllables, then the sensitivity to movement should be selective—it should obtain *only* in syllable count. Accordingly, when asked to judge the number of morphemes, signers should track the number of handshapes, rather than movements. Moreover, when presented with incongruent signs—signs in which the number of syllables is incongruent with the number of morphemes (e.g., in analogy to the English *cans* and *candy*)—signers should shift their response (one vs. two units) depending on the task—syllable vs. morpheme count.

Finding that, like spoken syllables, signed syllables are defined by sonority peaks could suggest that signed and spoken languages share an amodal phonological constraint. We next asked whether this principle is available to nonsigners, and whether it shapes their capacity to learn phonological rules in a new modality. To test this possibility, Experiments 2–4 compare the identification of these signs by three different groups of English speakers. Participants in all three groups had no previous experience of ASL, and they were provided with no feedback on their performance during the experimental sessions. These three experiments differed, however, with respect to the feedback provided to participants during the practice phase, presented prior to the experimental trials. Experiment 2 provided participants with no feedback at all, whereas Experiment 3 & 4 provided feedback in the practice session only (correct/incorrect messages). In Experiment 3, this feedback enforced the natural restriction on the structure of ASL syllables and morphemes, such that syllable structure was defined by movement (one movement per syllable) whereas morpheme structure was defined by handshape (one handshape per morpheme). Experiment 4 reversed the feedback, such that morpheme structure was defined by movement, whereas syllable structure was defined by handshape—an unnatural correspondence that is unattested in any language.

If experience (specifically, performance feedback) is necessary and sufficient to extract the restriction on syllable structure, then participants should be equally amenable to associate syllables with either movement or handshape, and their performance should faithfully mirror the feedback presented to them. Thus, absent feedback, in Experiment 2, people should show no preference to identify syllables according to the number of movements. And to the extent feedback is sufficient to induce syllable structure restrictions, then a natural correspondence on syllable structure (i.e., one movement per syllable) should be as easy to learn as an unnatural restriction (i.e., one handshape per syllable). In contrast, if the cross-linguistic preference to mark syllables by sonority/energy peaks results from an amodal phonological restriction, then nonsigners should spontaneously associate syllables with movement (in Experiment 2) and they should be primed to learn natural restrictions on syllable structure. Accordingly, participants should correctly associate movement with syllables, and learn to ignore it in counting morphemes (in Experiment 3). However, they might be unable to learn the reverse unnatural rule that requires them to ignore movement in counting syllables (in Experiment 4).

## Experiment 1: Deaf ASL Signers Selectively Attend to Both Syllables and Morphemes

### Results and discussion

To gauge the sensitivity of Deaf ASL signers to movement and handshape, we first examine the effects of movement and handshape on the syllable- and morpheme-count tasks, separately. To determine whether signers selectively use movement to define syllables, we next compared the two tasks in response to incongruent items (e.g., signs analogous to the English *candy*, with two syllables and one morpheme).

#### Syllable count

**Figure 3:**
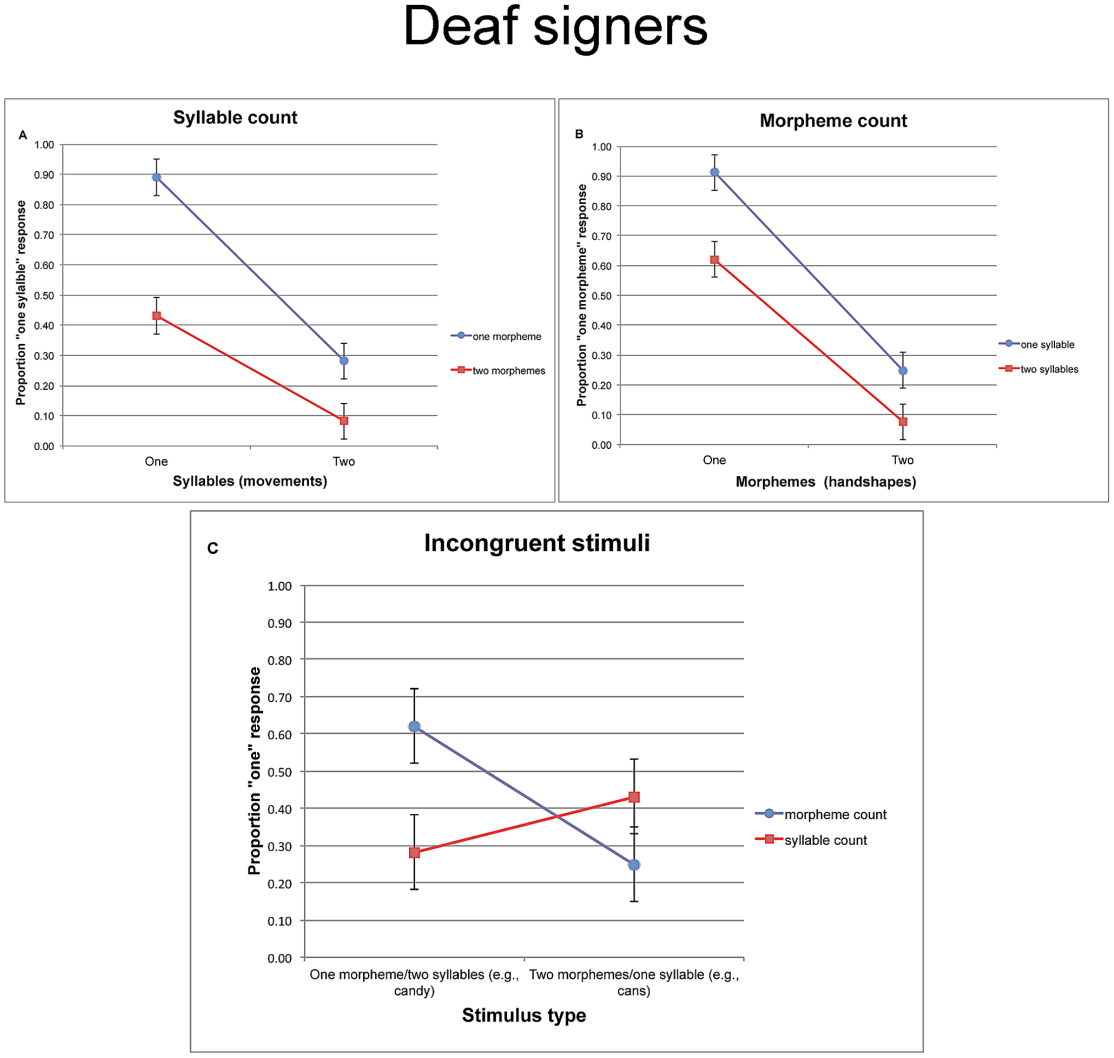
The proportion of “one” responses given by Deaf signers in Experiment 1 for the syllable count task (a), morpheme count task(b), and the incongruent trials taken from both tasks (c). Error bars are confidence intervals, constructed for the difference between the means.

Figure 3 depicts the proportion of “one syllable” responses in the syllable count task. An inspection of the means suggests that ASL signers were sensitive to the number of movements. Specifically, signs with one movement were more likely to elicit a “one syllable” response, and this was so irrespective of morphological structure (i.e., whether the sign had one handshape or two). We further tested the reliability of these observations using 2 syllable ×2 morpheme ANOVAs using both participants (F1) and items (F2) as random variables, with syllable (one movement vs. two) and morpheme (one handshape vs. two) as repeated measures (in this and all subsequent experiments, data were arcsine transformed). To assure that these results are not due to artifacts associated with binary data [66], we also submitted response accuracy data to a mixed-effects logit model, with syllable and morpheme, as fixed effects (sum-coded) and participants and items as random effects; the results are provided in Table 1.

**Table 1:** A Multilevel Logit analysis of Experiments 1–4.

Experiment	Condition	Fixed effects	β	SE	Z	*p*
Experiment 1	Syllable count	Syllable	1.84	0.1643	11.18	0.0001
		Morpheme	1.35	0.1608	8.42	0.0001
		Syllable x Morpheme	0.37	0.1586	2.33	.02
	Morpheme count	Morpheme	1.89	0.1273	14.92	.001
		Syllable	0.95	0.1208	7.89	.0001
		Morpheme x Syllable	0.21	0.1202	1.72	0.09
	Incongruent items	Task	1.91	0.2516	7.60	.0001
		Stimulus type	0.86	0.3378	2.56	0.02
		Task x Stimulus type	−2.95	0.3517	−8.39	0.0001
Experiment 2	Syllable count	Syllable	1.37	0.1494	9.18	0.0001
		Morpheme	0.82	0.1474	5.55	0.0001
		Syllable x Morpheme	0.00	0.1467	−0.02	0.98
	Morpheme count	Morpheme	0.69	0.1054	6.55	0.0001
		Syllable	0.91	0.1055	8.66	0.0001
		Morpheme x Syllable	0.10	0.1052	0.95	0.34
	Incongruent items	Task	−0.19	0.0776	−2.41	0.02
		Stimulus type	−0.37	0.1473	−2.53	0.02
		Task x Stimulus type	−0.13	0.0776	−1.68	0.1
Experiment 3	Syllable count	Syllable	1.64	0.1592	10.28	0.0001
		Morpheme	0.82	0.1580	5.18	0.0001
		Syllable x Morpheme	0.19	0.1577	1.19	.23
	Morpheme count	Morpheme	1.02	0.1183	8.65	0.0001
		Syllable	0.87	0.1184	7.63	0.0001
		Morpheme x Syllable	−0.11	0.1178	−0.90	.37
	Incongruent items	Task	−0.23	0.0835	−2.81	0.005
		Stimulus type	−0.32	0.1809	−1.78	0.08
		Task x Stimulus type	−0.47	0.0835	-5.61	0.0001
Experiment 4	Syllable count	Movement	1.10	0.1216	9.02	0.0001
		Handshape	1.15	0.1217	9.42	0.0001
		Movement x Handshape	−0.17	0.1213	1.39	.16
	Morpheme count	Handshape	0.80	0.1234	6.48	0.0001
		Movement	1.40	0.1241	11.31	0.0001
		Handshape x Movement	−0.08	0.1233	−0.65	.5
	Incongruent items	Task	0.31	0.0774	4.03	0.0001
		Stimulus type	−0.29	0.1288	−2.27	0.03
		Task x Stimulus type	0.31	0.0774	4.03	0.0001

These analyses yielded significant main effects of syllable (F1(1, 14) = 59.42, MSE = .13, p<.0001; F2(1, 12) = 199.48, MSE = .02, p<.0001), morpheme (F1(1, 14) = 41.56, MSE = .08, p<.0001; F2(1, 12) = 72.58, MSE = .04, p<.0003) and a reliable syllable x morpheme interaction (F1(1, 14) = 19.63, MSE = .05, p<.0006; F2(1, 12) = 45.29, MSE = .01, p<.003).

The effect of syllable structure shows that signers were reliably more likely to give a “one syllable” response to novel signs with one movement relative to signs with two movements. Syllable count, however, was modulated by the number of handshapes. Tukey HSD tests showed that participants were reliably less likely to give a correct “one syllable” response to monosyllabic signs that were morphologically complex (p<.0002 by participants and items) relative to monosyllabic monomorphemic signs, and they were also slightly more likely to give correct disyllabic responses to disyllables that are morphologically complex relative to those that are morphologically simple (this latter trend was only marginally significant; p>.12, p<.005, by participants and items, respectively). To use and English analogy, signers were less likely to correctly classify *cans* as monosyllabic compared to the monomorphemic *can*, and they were also slightly more likely to classify *candies* as disyllabic compared to the monomorphemic *candy*. This effect suggests that the handshape complexity (i.e., a sequence of two phonologically distinct handshapes) of bimorphemic signs interfered with their identification as monopartite at the phonological level. Nonetheless, Tukey HSD tests demonstrated that people were sensitive to the number of movements irrespective of morphological complexity—for both monomorphemic (p<.0002 by participants and items) and bimorphemic items (p<.0007, by participants and items).

#### Morpheme count

While syllable count was sensitive to the number of movements, morpheme count tracked the number of handshapes. The proportion of “one morpheme” responses is presented in Figure 3. The 2 morpheme ×2 syllable ANOVAs yielded a reliable main effect of morpheme (F1(1, 14) = 52.57, MSE = .22, p<.0001; F2(1, 12) = 227.45, MSE = .03, p<.001), syllable (F1(1, 14) = 15.79, MSE = .11, p<.002; F2(1, 12) = 75.51, MSE = .03, p<.0001) and a reliable morpheme x syllable interaction (F1(1, 14) = 7.07, MSE = .05, p<.02; F2(1, 12) = 20.82, MSE = .02, p<.0007).

These analyses show that signers were more likely to identify signs with two handshapes as morphologically complex. Nonetheless, their morphological sensitivity was attenuated by conflicting syllabic information, resulting in a reliable interaction. Tukey HSD tests showed that correct monomorphemic responses were more likely for monosyllables (e.g., the English *can*) compared to disyllables (e.g., *candy*, p<.0003, by participant and items), and they were also slightly more likely to identify *candies* as bimorphemic (relative to *cans*), although this latter trend was not reliable (p>.14, p<.04, by participants and items, respectively). This effect of syllable suggests that the movement complexity of disyllabic signs interfered with their identification as monopartite at the morphological level—a phenomenon analogous to the interference of handshape complexity with syllable count. Nonetheless, people were sensitive to the number of handshapes of both monosyllabic (p<.0002, by participants and items) and disyllabic signs (p<.0002, by participants and items).

#### Responses to incongruent items

The results presented so far suggest that syllable- and morpheme count are each constrained by the variable of interest—movement vs. handshape respectively. Each task, however, was also modulated by interference from the orthogonal dimension (e.g., responses to monosyllabic items were impaired by handshape complexity). Accordingly, these data cannot establish which dimension was used to define syllables—whether syllables were in fact defined by sonority peaks (e.g., movements), or by the conjunction of movement and handshape complexity. The comparison of responses in these two tasks to incongruent signs directly addresses this issue (Figure 3).

Incongruent items (e.g., items analogous to *cans*, *candy*) exhibit a mismatch between the number of syllables and the number of morphemes, so their categorization dissociates these competing dimensions. A monosyllabic response to *cans*-type items suggests that syllables are defined by movement, whereas their categorization as bimorphemic would demonstrate that morphemes are determined by handshape. If syllables are defined by movement, then responses to incongruent items should shift depending on the task (syllable vs. morpheme count). To the extent signers can further selectively focus their attention on movement and ignore conflicting handshape information, then syllable count should exhibit a higher rate of monopartite responses to *cans-* relative to *candy*-type items, whereas morpheme count should yield the opposite pattern.

A separate 2 task (morpheme vs. syllable count) ×2 stimulus type (monomorphemic disyllables vs. bimorphemic monosyllables) analyses of incongruent items indeed yielded a significant effect of task (F1(1, 14) = 6.13, MSE = .03, p<.03; F2(1, 12) = 6.59, MSE = .02, p<.03) and a marginally significant effect of stimulus type (F1(1, 14) = 2.11, MSE = .15, p<.17; F2(1, 12) = 3.69, MSE = .06, p<.08). Crucially the task x type interaction was reliable (F1(1, 14) = 11.16, MSE = .20, p<.005; F2(1, 12) = 88.77, MSE = .01, p<.0001).

Planned comparisons showed that participants were reliably more likely to identify items like *candy* (monomorphemic disyllables) as monopartite in the morpheme count compared to the syllable count task (t1(14) = 3.10, p<.008; t2(12) = 10.35, p<.0001), whereas items like *cans* (monosyllabic bimorphemes) exhibited an opposite (nonsignificant) pattern (t1(14) = 1.62, p<.13; t2(12) = 5.34, p<.0002). Not only did signers shift their responses across tasks but also they were able to selectively shift their attention within each procedure. Specifically, signers were reliably more likely to provide a monomorphemic response to signs analogous to *candy* than to signs like *cans* (t1(14) = 3.23, p<.007; t2(12) = 11.56, p<.0001), whereas the opposite (nonsignificant) pattern obtained in the syllable count, that is, a higher monosyllabic response to *cans* than *candy* (t1(14) = 1.49, p<.16; t2(12) = 4.13, p<.002). The rate of monosyllable responses nonetheless differed from chance for *candy-*type items (M = .28, t1(14) = 5.08, p<.0002; t2(12) =  −3.59, p<.004; for *cans*-type items: M = .43, t1(14)<1; t2(12) = 2.26, p<.05), whereas morpheme-count responses exceeded chance for *cans*-type items (M = .25, t1(14) =  −4.28, p<.0008; t2(12) =  −7.27, p<.0001; for *candy*-like items: M = .62, t1(14) = 1.31, p<.22; t2(12) = 3.19, p<.008).

The superior ability to count the number of constituents (syllables and morphemes) in bipartite items might be due to the fact that their monopartite counterparts are unmarked (i.e., unspecified) for the relevant linguistic structure (for related experimental evidence, see [67]). Nonetheless, the overall level of categorization of incongruent items was far from perfect. This finding is hardly surprising, as these stimuli present a tall order for the evaluation of linguistic rules—not only do they test the representation of productive rules, but they further gauge signers' ability to selectively attend to the relevant linguistic dimension (e.g., movement) in the face of conflicting information from the other (e.g., handshape). Finding that signers reliably shifted their responses to the same incongruent items depending on the task, and that they selectively attended to movement for the purpose of syllable count suggests that they encode two productive linguistic principles. One rule selectively defines syllables (but not morphemes) by movement; another constrains morphemes to a single contrastive handshape. While past research has shown that signers rely on movement in segmentation [56], the present results provide the first experimental demonstration that signers distinguish syllables and morpheme-like units, and they constrain their structure by productive rules that apply to novel signs.

## Experiment 2: Nonsigners Spontaneously Rely on Movement in Segmenting Signs

Our findings that sonority peaks define syllables in ASL converge with past experimental and linguistic results from spoken language [7], [8], [16]–[19], [34]–[42] and the linguistic evidence from sign languages [14], [15], [20]–[26] to suggest that sonority constrains the structure of the syllable across modalities. Why do different languages converge on this restriction?

One possibility is that signed and spoken languages independently developed distinct restrictions on syllable structure. But on an alternative account, the convergent design reflects a common amodal phonological principle. The hallmark of an amodal principle is that it is selective in its definition, but broad in its application. The sonority restriction on syllable structure potentially meets both criteria. It is sufficiently narrow in its application to syllables (but not to morphemes) to suggest a specific linguistic rule (rather than a generic cognitive restriction), but its broad application to speech and sign shows that the description of the rule is sufficiently abstract to apply across modalities. To evaluate this possibility, we next asked whether the restriction on syllable structure of signed languages might be available to nonsigners who have had no previous experience with a sign language.

Experiment 2 thus administers the syllable- and morpheme-count tasks to English speakers. If participants know that all syllables—signed and spoken—require sonority/energy peak, then they might be inclined to favor movement (a peak of visual energy) over handshape as a clue for defining syllable-like units. Nonsigners, however, might be unable to identify ASL morphemes, as they lack evidence for the phonological restrictions on morphological structure (one group of selected fingers per morpheme). Since nonsigners can only extract syllable-like units, they might invariably rely on movement, irrespective of whether they are asked to count syllables or morphemes. Whether the exclusive focus on movement is due to the visual salience of movement or its linguistic role in defining syllabic units is a question we address in subsequent experiments.

### Results and discussion

**Figure 4:**
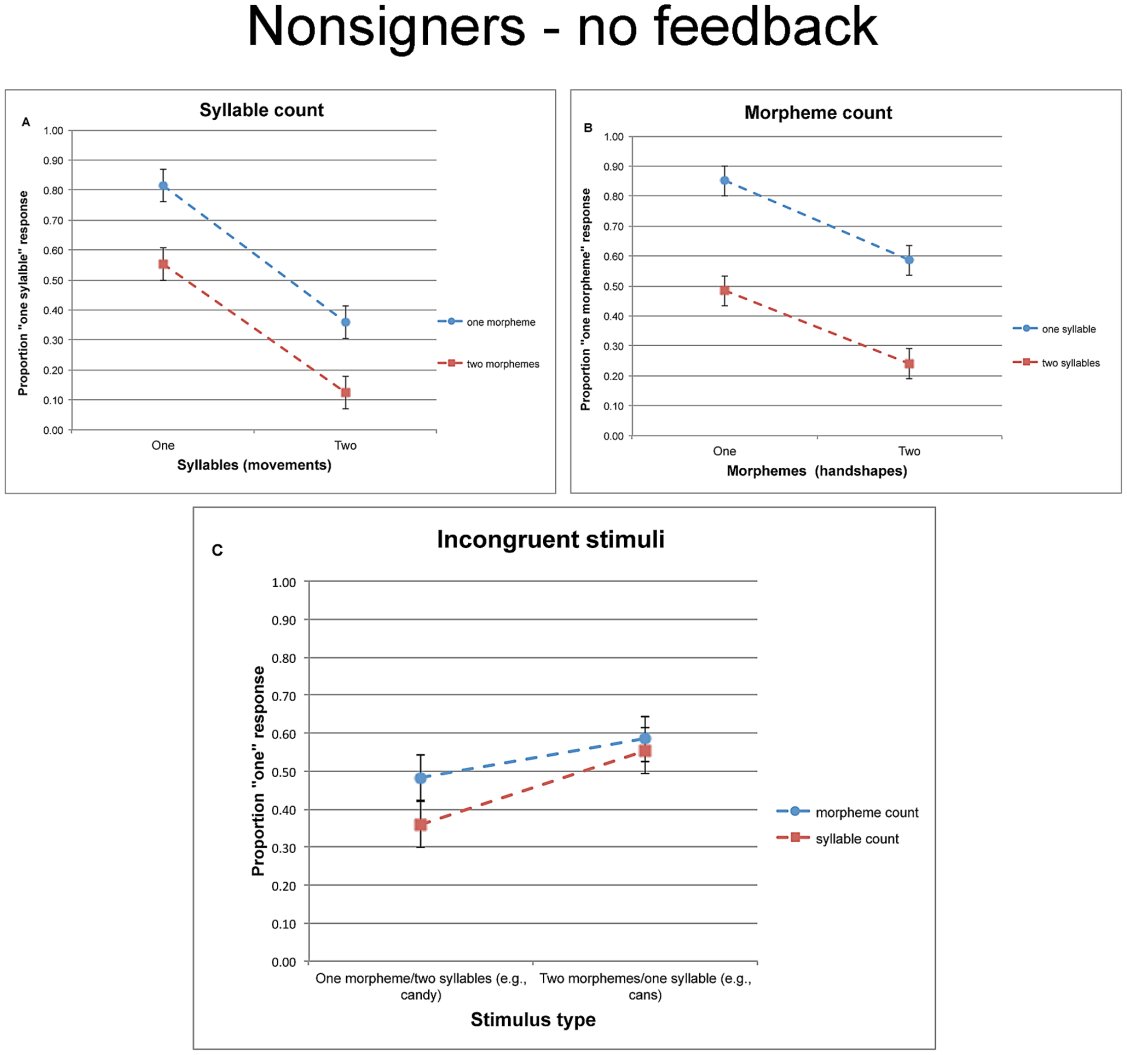
The proportion of “one” responses given by nonsigners in Experiment 2 (without feedback) for the syllable count task(a), morpheme count task (b), and the incongruent trials taken from both tasks (c). Error bars are confidence intervals, constructed for the difference between the means.

An inspection of the means (see Figure 4) suggests that nonsigners were sensitive to both movement and handshape information. We first probe the effects of these two dimensions on syllable- and morpheme-count. To examine whether nonsigners specifically favored movement over handshape in defining syllables, we next examine responses to incongruent items.

#### Syllable count

A 2 syllable (one vs. two movements) ×2 morpheme (one vs. two handshapes) analysis of the syllable task yielded reliable main effects of both syllable (F1(1, 14) = 81.69, MSE = .06, p<.001; F2(1, 12) = 87.79, MSE = .04, p<.0001) and morpheme (F1(1, 14) = 53.11, MSE = .03, p<.0001; F2(1, 12) = 22.68, MSE = .06, p<.0006). The interaction was marginally significant (F1(1, 14) = 6.39, MSE = .02, p<.03; F2(1, 12) = 1.17, MSE = .06, p<.31). Tukey HSD tests confirmed that participants were reliably more likely to identify novel signs with one movement as monosyllabic, and this was the case regardless of whether those signs were morphologically simple (p<.0002, by participants and items) or complex (p<.004, by participants and items).

#### Morpheme count

Similar analyses on performance in the morpheme count task yielded reliable main effects of morpheme (F1(1, 14) = 16.93, MSE = .11, p<.0002; F2(1, 12) = 17.59, MSE = .09, p<.0002) and syllable (F1(1, 14) = 29.04, MSE = .14, p<.0001; F2(1, 12) = 182.93, MSE = .02, p<.0001). The interaction was only marginally significant (F1(1, 14) = 4.91, MSE = .023, p<.05; F2(1, 12) = 1.86, MSE = .06, p<.20). Once again, participants were reliably more likely to identify signs with one handshape as monomorphemic compared to signs with two handshapes, and this was the case regardless of whether the sign was monosyllabic (p<.003, Tukey HSD test, by participants and items) or disyllabic (p<.06, Tukey HSD test, by participants and items).

#### Incongruent items

While these results demonstrate that nonsigners can spontaneously track both movement and handshape, these observations do not determine the linguistic functions of these dimensions. A selective reliance on movement as a cue for syllables should result in a shift in responses to incongruent items depending on the task—syllable vs. morpheme count. Unlike signers, however, the responses of nonsigners to incongruent signs were not modulated by the task (Figure 4).

The 2 task (morpheme vs. syllable count) ×2 stimulus type (monomorphemic disyllables vs. bimorphemic monosyllables) ANOVAs did not yield a reliable interaction (F1(1, 14)<1; F2(1, 12) = 1.27, MSE = .02, p<.29). However, the main effect of task was significant (F1(1, 14) = 3.53, MSE = .09, p<.05; F2(1, 12) = 5.12, MSE = .02, p<.05). Moreover, nonsigners were sensitive to the structure of these stimuli, as they were more likely to yield a monopartite response to *cans* relative to *candy*-type items (across tasks). The main effect of stimulus type was marginally significant in the ANOVAs (F1(1, 14) = 5.15, MSE = .11, p<.04; F2(1, 12) = 3.88, MSE = .12, p<.08), and it was fully reliable in the logit model (see Table 1). Syllable-count responses reliably differed from chance for *candy*- (M = .36, t1(14) = −2.53, p<.03; t2(12) = −2.89, p<.02), but not for *cans-*type items (M = .55, t1(14)<1; t2(12) = 1).

Taken as a whole, the findings of Experiment 2 suggest that nonsigners encode both movement and handshape, and when provided with incongruent signs, nonsigners are biased to base their syllable count on movement despite conflicting handshape information. However, their reliance on movement was not selective, as the classification of incongruent monosyllables and disyllables did not change reliably as a function of the task—morpheme vs. syllable count.

These findings are open to two distinct interpretations. One possibility is that nonsigners fail to extract signed syllables—they only encode visual units (i.e., the units used by the visual system to encode its inputs, generally), not linguistic phonological constituents; the alternative is that they *only* extract syllabic units. Since nonsigners lack knowledge of ASL morpheme structure constraints (i.e., they have no evidence that ASL morphemes require one handshape), the only unit available to them is the syllable, and consequently, responses to incongruent items are invariably guided by movement. This latter explanation assumes that nonsigners possess an amodal restriction on syllable structure; the former assumes that the restriction on syllable structure is modality-specific. The following experiments attempt to adjudicate between these possibilities.

## Experiment 3: Nonsigners Can Learn to Partly Ignore Movement in Counting Morphemes

Why are nonsigners sensitive only to syllable-like units? Do they extract linguistic constituents, defined by sonority/energy peaks, or are they only guided by the overall visual salience of movement? To dissociate between these explanations, Experiment 3 examines whether nonsigners might learn to selectively apply this phonological condition to syllables, but ignore it in defining morphemes. To this end, we repeated the design and procedure of Experiment 2, except that now, the practice session provided participants with brief feedback on their performance, identical in its extent to the feedback provided to signers in Experiment 1 (feedback on accuracy in response to 8 practice trials with real signs and 8 practice trials with novel signs). Because the syllable structure restriction implied by this feedback is attested in ASL and practically every known sign language, we refer to it as a natural phonological rule.

While such brief feedback is clearly insufficient to extract the full linguistic structure of signs, it might nonetheless allow nonsigners to discover the phonological restriction on handshape, and use it to extract morpheme-like units. Those units may not necessarily correspond to morphemes (i.e., abstract categories of form-meaning pairings) and their extraction may not be fully reliable. Our question here is whether nonsigners may nonetheless learn to ignore movement in segmenting those units. That is, will nonsigners now selectively rely on movement in defining syllables, but not morphemes? Whether feedback itself is sufficient to promote such learning is a question we leave for the next experiment.

### Results and discussion

**Figure 5:**
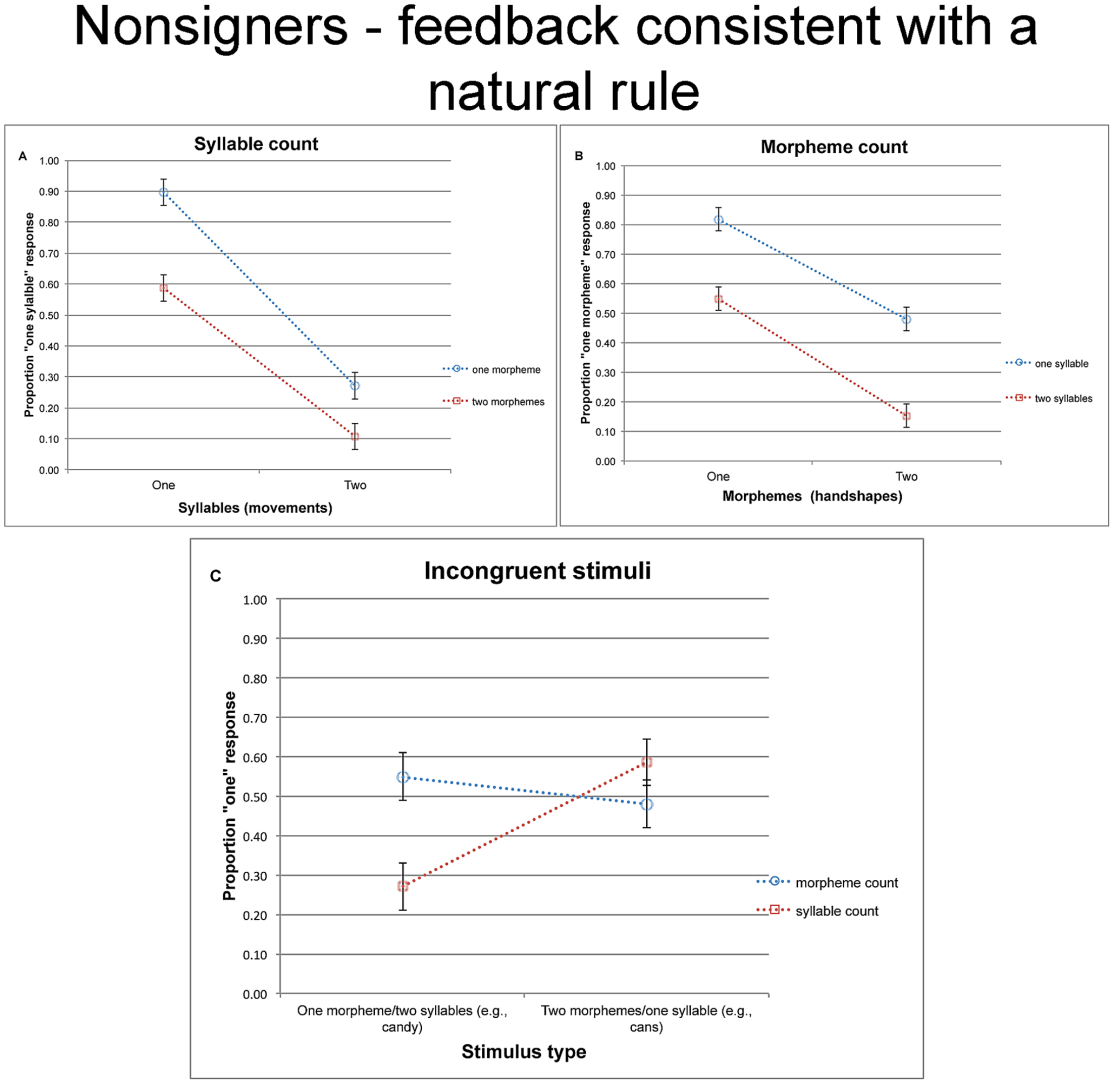
The proprtion of “one” responses given by nonsigners in Experiment 3 (with feedback consistent with the natural phonological association of syllables and movement) for the syllable count task (a), morpheme count task (b), and the incongruent trials taken from both tasks (c). Error bars are confidence intervals, constructed for the difference between the means.

The responses of nonsigners in the syllable- and morpheme-count tasks are presented in Figure 5. An inspection of the means suggests that nonsigners were sensitive to both movement and handshape. However, responses to each of these dimensions (e.g., movement) were impaired by incongruency from the other dimension (e.g., handshape). These conclusions are borne out by the separate analyses of the two tasks.

#### Syllable count

A 2 syllable (one vs. two movements) ×2 morpheme (one vs. two handshapes) ANOVA yielded significant effects of syllable (F1(1, 14) = 118.93, MSE = .08, p<.0001; F2(1, 12) = 173.05, MSE = .04, p<.0001), morpheme (F1(1, 14) = 43.53, MSE = .05, p<.0002; F2(1, 12) = 19.50, MSE = .08, p<.0009), and their interaction (F1(1, 14) = 21.62, MSE = .04, p<.0004; F2(1, 12) = 4.47, MSE = .08, p<.06). Tukey HSD tests confirmed that nonsigners were reliably sensitive to the number of movements for both monomorphemic (Tukey HSD tests: p<.0003, by participants and items) and bimorphemic signs (Tukey HSD tests: p<.0003, by participants and items).

#### Morpheme count

The 2 morpheme ×2 syllable ANOVAs on the morpheme count responses yielded significant main effects of morpheme (F1(1, 14) = 34.71, MSE = .10, p<.0004; F2(1, 12) = 48.07, MSE = .06, p<.0001) and syllable (F1(1, 14) = 27.02, MSE = .08, p<.0002; F2(1, 12) = 73.29, MSE = .02, p<.0001). The interaction was not significant (both F<1).

#### Response to incongruent trials

The separate analyses of the syllable- and morpheme count tasks confirm that nonsigners in the present experiment extract both movement and handshape information—results that are in line with the findings of Experiment 2. Of primary interest is whether the brief feedback provided to nonsigners in the practice session allowed them to discover morpheme-like units (defined by handshape), and distinguish them from syllables—units defined by movement. To address this question, we now turn to the incongruent conditions.

An inspection of the means (Figure 5) suggests that people shifted their response to incongruent stimuli depending on the task. The 2 task (morpheme vs. syllable count) ×2 stimulus type (monomorphemic disyllables vs. bimorphemic monosyllables) ANOVAs on incongruent trials yielded a reliable interaction (F1(1, 14) = 16.47, MSE = .06, p<.002; F2(1, 12) = 38.97, MSE = .02, p<.0001). The main effects of task (F1(1, 14) = 5.36, MSE = .03, p<.04; F2(1, 12) = 3.38, MSE = .03, p<.10) and stimulus type (F1(1, 14) = 2.29, MSE = .10, p<.16; F2(1, 12) = 2.21, MSE = .13, p<.17) were not significant

Planned contrasts demonstrated that participants were reliably more likely to classify disyllabic-monomorphemic stimuli (e.g., the equivalent of *candy*) as monopartite in the morpheme-count task compared to the syllable count task (t1(14) = 4.24, p<.002; t2(12) = 5.98, p<.001), whereas the reverse trend emerged for monosyllabic-bimorphemic stimuli (isomorphic to *cans*, t1(14) = 1.61, p<.13; t2(12) = 2.85, p<.02). Moreover, nonsigners were able to track the number of both syllables and morphemes. Accordingly, they were reliably more likely to give a monosyllabic response to *cans-* than to *candy*-like stimuli (t1(14) = 4.63, p<.0001; t2(12) = 7.16, p<.0001), and responses to *candy*-like stimuli were further significantly different from chance (for *candy*: M = .27, t1(14) = −4.90, p<0003; t2(12) = −3.87, p<.003; for *cans*: M = .59, t1(14) = 2.03, p<.07; t2(12) = 1.40, p<.19). In contrast, participants in the morpheme-count task gave a numerically higher rate of monopartite response to *candy* than to *cans*, but this trend was not significant (t1(14) = 1.47, p<.17; t2(12) = 1.67, p<.13), and the classification of these stimuli did not differ from chance (for *candy*: M = .55, t1(14)<1; t2(12) = 1.27, p<.23); for *cans* M = .48, both t<1).

## Experiment 4: Can Nonsigners Learn to Ignore Movement in Counting Syllables?

The results presented so far suggest that signers and nonsigners favor movement over handshape as a cue for syllable structure. Absent any experience with signs, nonsigners in Experiment 2 spontaneously segmented signs by movement, and given minimal evidence for the phonological restriction on morphemes (one handshape per morpheme), nonsigners in Experiment 3 learned to partly ignore movement in defining morpheme-like units. While these morpheme-like units were not reliably identified nor did they necessarily correspond to form-meaning pairings, they nonetheless clearly differed from syllables. This divergence shows that nonsigners can learn to *selectively* rely on movements in defining syllables, in a manner comparable to fluent Deaf ASL signers. Such selectivity, however, was only evident given relevant experience with signs—either exposure to ASL (for signers) or brief practice (for nonsigners). Accordingly, one wonders whether experience might be also sufficient to explain these findings.

To address this issue, we next examined whether people are constrained with respect to their ability to learn from linguistic experience with signs. We reasoned that if feedback is necessary and sufficient to promote the induction of syllable structure, then people's capacity to learn the natural restriction on syllable structure (one sonority peak per syllable)—a restriction active in every natural language—should not differ from their capacity to learn an unnatural restriction that is unattested in phonological systems (one handshape per syllable). Conversely, if people are inherently biased to define syllables by sonority/energy peaks, then they might fail to learn unnatural phonological restrictions on the syllable.

To test this possibility, Experiment 4 administers the syllable- and morpheme- count tasks to a new group of English speaking participants. The materials, design and procedure are identical to those used in our previous experiments, and as in Experiment 3, we also provided participants with the opportunity to learn by presenting them with feedback on their performance in the practice session only (identical in its extent to the feedback provided in Experiments 1–2). Critically, however, the feedback was now reversed, such that syllable structure was paired with handshape, whereas morpheme structure was linked to movement. This change was implemented by reversing the feedback on incongruent trials, such that signs with two movements and one handshape (*candy-*type items) were classified as monosyllabic, whereas those with two handshapes and one movement (*cans-*type items) were presented as monomorphemic (the feedback on congruent trials remained unchanged).

In view of people's exquisite sensitivity to statistical structure, we expect them to alter their responses in accord with the contingencies presented to them. Consequently, performance in the incongruent conditions should now reverse: participants should be more likely to interpret *candy*-type items as having two parts in the morpheme- compared to the syllable count task, whereas the opposite should occur for *cans*-type items. Of interest is what principle was induced by participants—whether they learned to associate morpheme-like units with movement (a restriction that is not universal, but certainly attested in many languages), or whether they effectively learned to define syllables by handshape—an unnatural phonological restriction.

### Results and discussion

#### Syllable count

**Figure 6:**
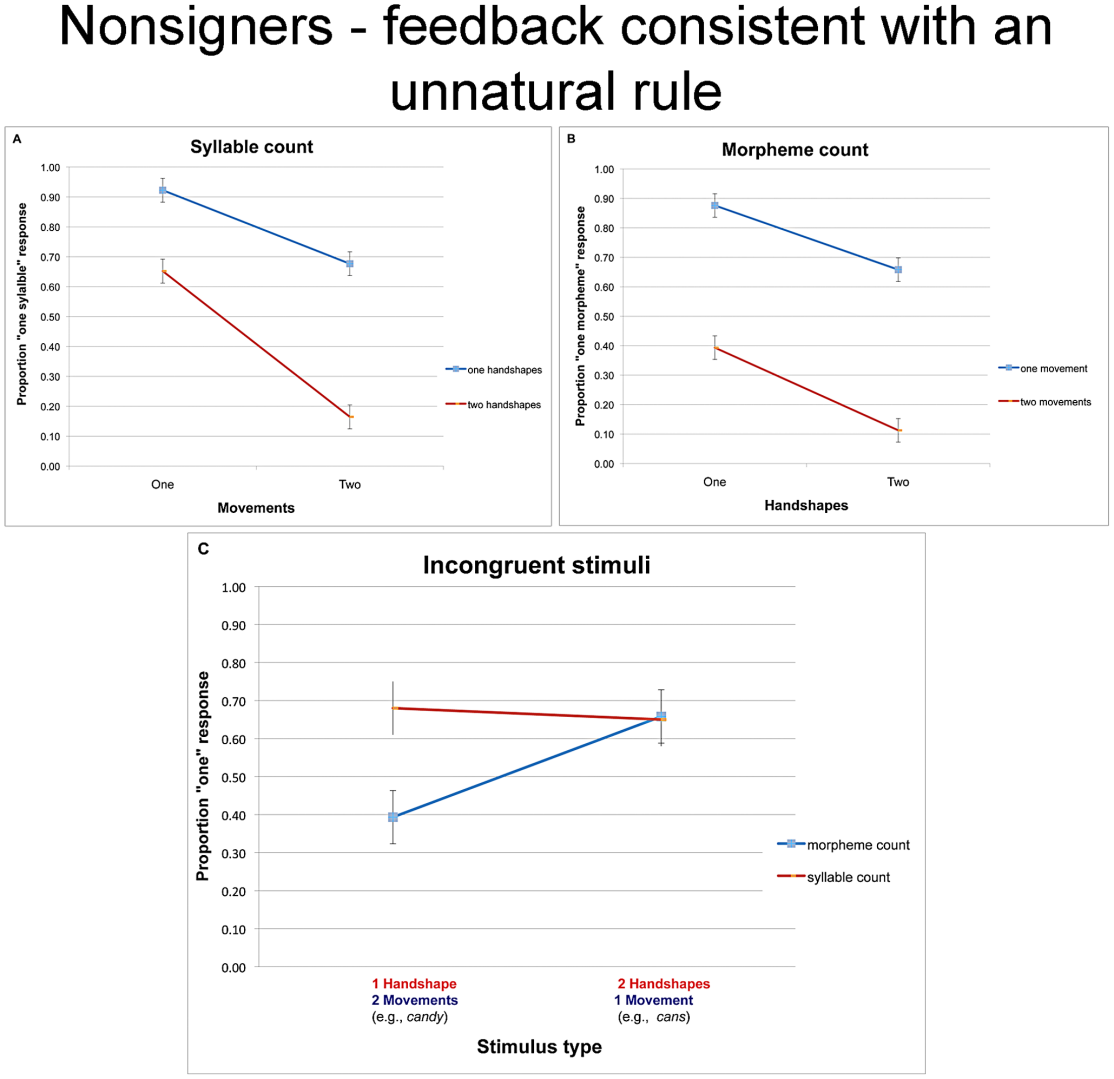
The proportion of “one” responses given by nonsigners in Experiment 4 (with feedback suggesting an unnatural phonological association of syllables and handshape) for the syllable count task (a), morpheme count task (b), and the incongruent trials taken from both tasks (c). To clarify the effect of learning from feedback, we indicate the expected responses, color-coded by task. Specifically, syllable count responses (in red) should depend on the number of handshapes; morpheme count (in blue) should depend on the number of movements. Error bars are confidence intervals, constructed for the difference between the means.

Figure 6 plots the syllable count responses as a function of movement and handshape (because the feedback given to participants defines syllable structure by handshape, rather than movement, we now do not describe our independent variables as “syllable” and “morpheme”). An inspection of the means suggests that, despite the reversal in feedback, participants remained sensitive to the number of movements and handshapes. Accordingly the 2 movement ×2 handshape analyses on the syllable count task yielded reliable main effects of movement (F1(1, 14) = 76.42, MSE = .05, p<.0001; F2(1, 12) = 78.02, MSE = .05, p<.0001) and handshape (F1(1, 14) = 47.46, MSE = .09, p<.0001; F2(1, 12) = 76.30, MSE = .05, p<.0001). The interaction was not significant (F1(1, 14) = 2.14, MSE = .04, p<.17; F2(1, 12)<1).

#### Morpheme count

Similar analyses performed on the morpheme count task likewise yielded reliable effects of handshape (F1(1, 14) = 32.21, MSE = .07, p<.0001; F2(1, 12) = 21.26, MSE = .07, p<.0006) and movement (F1(1, 14) = 75.37, MSE = .10, p<.0001; F2(1, 12) = 170.50, MSE = .03, p<.0001). The interaction was not significant (F1(1, 14) = 2.37, MSE = .04, p<.15; F2(1, 12)<1).

#### Responses to incongruent items

The results reported so far suggest that nonsigners were able to track both the number of movements and handshapes. Our main interest, however, is whether they learned to shift their reliance on these dimensions as cues for syllable vs. morpheme structure given the feedback they were provided. The analysis of the incongruent conditions (see Figure 6) appears to support for this possibility.

The 2 task (syllable vs. morpheme) ×2 stimulus type (monomorphemic disyllables vs. bimorphemic monosyllables) ANOVAs yielded a reliable interaction (F1(1, 14) = 8.13, MSE = .06, p<.02; F2(1, 12) = 14.67, MSE = .03, p<.003). Planned comparisons showed that responses to *cans*-type items (bimorphemic monosyllabic) did not differ in the two tasks (both t<1), whereas the tasks did shift responses to *candy-*type items (disyllabic monomorphemic). Remarkably, participants were now *more* likely to identify *candy-*type items as monopartite in the syllable- compared to the morpheme-count task (t1(14) = 4.17, p<.002; t2(12) = 5.21, p<.001). Morpheme-count responses to these incongruent items were also reliably modulated by the number of movements (t1(14) = 3.76, p<.003; t2(12) = 4.95, p<.001).

The shift in response to incongruent items demonstrates that participants were able to learn from the reverse feedback provided to them. While in Experiment 3, items like *candy* were more likely to elicit monopartite responses in the morpheme-count relative to the syllable-count task, here, this pattern was now reversed.

At first blush, this finding would appear to suggest that participants learned to associate syllables with handshape. But a closer inspection suggests that this interpretation is unlikely (to clarify the role of feedback, Figure 6 indicates the response expected by the feedback, color-coded for task). First, responses in the syllable-count task were utterly unaffected by stimulus type—monosyllabic responses were no more likely to signs with a single handshape relative to those with two handshapes (both t<1). The insensitivity of syllable count to the number of handshapes stands in marked contrast to its systematic modulation by the number of movements, documented in the three previous experiments. Moreover, while participants reliably learned to classify *candy*-type items (i.e., disyllabic monomorphemes) as phonologically monopartite (M = .67, t1(14) = 2.95, p<.02; t2(12) = 4.53, p<.0007), they were utterly unable to classify signs like *cans* (monosyllabic bimorphemes) as bipartite. In fact, participants systematically classified such items as monopartite, and their tendency to do so differed reliably from chance (M = 0.65, t1(14) = 2.78, p<.02; t2(12) = 3.37, p<.006). This response is consistent with their classification in all previous experiments, and inconsistent with the feedback provided to them.

Participants' failure to base their syllable-count on the number of contrastive handshapes, and their persistent classification of signs with two handshapes as monosyllabic shows that they were unable to effectively learn an unnatural rule that links syllable to handshape. Instead, participants seem to have acquired two natural correspondences. Their systematic “monopartite” responses in the syllable count could reflect the encoding of prosodic feet—a higher-level prosodic constituent. Since *candy*- and *cans-*type items are both one-footed, they are invariably identified as prosodically monopartite. In contrast, the sensitivity of morpheme-count to the number of movements suggests the encoding of syllable-like units. This could either occur because participants in this condition effectively counted syllables (defined by movement), rather than morphemes. Alternatively, they might have learned a rule that defines morpheme-like units by the number of movements. While morphological rules do not necessarily affect the number of syllables, many morphological processes do so (e.g., the added morphemes in *UNdo*, *DISlike, parkING*), hence, the link between morphemes and sonority/energy peaks is widely attested (albeit not systematically required). Although the particular strategies acquired by participants in this experiment are open to multiple interpretations, it is clear that they did not define syllable-like units by handshape.

Participants' resistance to induce an unnatural rule that associates syllables with contrastive handshape, coupled with their spontaneous capacity to base syllable count on movement (in Experiment 3) are both consistent with the possibility that nonsigners impose restrictions on the sonority sequencing of syllables, and these restrictions constrain their ability to learn from experience.

## General Discussion

The present research investigated whether signed and spoken languages share common amodal restrictions on syllable structure. Across languages, syllables require a single sonority peak whereas morphemes are not so constrained. Here, we asked whether this amodal principle forms part of the linguistic competence of signers and nonsigners. Experiment 1 showed that signers distinguish syllables from morphemes in novel signs. These results demonstrate that signers encode a productive linguistic restriction on syllable structure, distinct from the restriction on morphemes. We next asked whether similar preferences are available to nonsigners who lack any previous experience with a sign language. Experiment 2 showed that, absent any feedback, nonsigners can spontaneously track both movement and handshape information, but they favor movement over handshape as a cue for the segmentation of incongruent signs. Nonsigners, however, were unable to extract ASL morphemes, so it was impossible to determine whether their reliance on movement is due to a linguistic bias to define syllables by movements or a purely visual preference. To adjudicate between these possibilities, Experiment 3–4 examined the propensity of nonsigners to learn phonological restrictions on syllable structure.

Given minimal implicit feedback, nonsigners in Experiment 3 were able to rapidly learn a natural phonological rule that links syllables to movement, and they were also now able to partly ignore movement in defining morpheme-like units. But remarkably, nonsigners were unable to learn to ignore movement in defining syllables (in Experiment 4). This was not due to an across-the-board failure to learn, as participants in this experiment markedly altered their morpheme count in response to feedback. Nonsigners' failure to learn the syllable-handshape link is also not due to a general insensitivity to handshape, as this factor reliably modulated their performance in all three experiments. Finally, nonsigners' inability to link syllables and handshape is not due their overall inability to ignore incongruent movement information, as they were at least partly able to do so in Experiment 3. We thus conclude that nonsigners were biased to associate signed syllables with movement. This result converges with past findings to suggest that signed [14], [15], [20]–[26] and spoken [7], [8], [16], [18], [19], [34]–[42] languages are constrained by a common restriction that requires a syllable to exhibit a single sonority/energy peak.

What is the reason for this convergence? Finding that distinct naturally occurring systems share structural properties does not, in and of itself, demonstrate that the shared feature is amodal. For example, sign languages, music and the visual system all have the capacity to encode hierarchical structure, but this convergence could reflect a generic computational mechanism that is independently deployed in different areas. Truly amodal principles, in contrast, are ones that are both narrowly defined, and consequently, likely to rely on domain-specific knowledge, but their description is sufficiently abstract to apply broadly, across modalities. The sonority restriction on syllable structure arguably meets both conditions. The requirement for a sonority/energy peak is selectively applied to constrain the structure of the syllable (but not the morpheme), and our experimental findings show that people enforce this restriction in a specific, targeted manner. Nonetheless, knowledge of this restriction in one linguistic modality (spoken languages) spontaneously extends to another (sign). We thus conclude that the association of signed syllables with movement presents an amodal linguistic preference. Moreover, this principle is available to signers and nonsigners alike, irrespective of their experience with sign languages.

The documentation of amodal phonological principles is somewhat unexpected given the intimate link between the structure of the phonological system and its phonetic channel [68]. Our findings do not undermine this fact. While labial consonants only emerge in oral languages and handshape is the exclusive property of manual systems, some broader aspects of design are shared. The existence of such shared amodal linguistic restrictions also does not negate the undeniable role of linguistic experience in the identification of signs. For example, four month old infants are sensitive to the phonetic handshape categories of ASL irrespective of linguistic experience [51], [52], but at fourteen months of age, this distinction is maintained only in signing infants [52], but not in nonsigning infants [51] and adults [48]. The presence of shared biases is not inconsistent with these facts. Amodal linguistic restrictions are not expected to render the structure of sign languages patent to nonsigners. Rather, they might constrain the range of linguistic representations computed by people to signs. They may also help explain the widespread cross-linguistic tendency to favor sonorous elements (e.g., vowel, movement) as cues for syllable structure.

Our findings leave several open questions. While the results suggest that signers and nonsigners distinguish syllable- from morpheme-like units, our present findings do not allow us to determine the precise nature of those linguistic constituents. Because ASL morphemes are subject to a phonological co-occurrence restriction, participants (signers and nonsigners) could have well represented morpheme-like units without specifically encoding them as form-meaning pairings. Similar questions apply to the representations of syllables, as it is unclear from these findings whether the units extracted by participants are phonological syllables constrained by sonority, or phonetic syllable-like units that require a peak of phonetic energy. Either way, it is evident that, across modalities, syllable-like (but not morpheme-like) units require a single sonority/energy peak.

Our findings also do not speak to the crucial question of whether the principles that define these units are experience-dependent. It is in fact conceivable that English speakers might have modeled the restrictions on signed syllables from their experience with their language. While it is unlikely that participants relied on conscious analogical reasoning, as our subsequent studies found that people have great difficulties to deliberately analogize the structure of signs to English examples (e.g., “what complex sign relates to its base as *kidney* to *kid*”), it is quite possible that participants in the present experiments relied on implicit knowledge of English phonology. Nonetheless, speakers have been shown to apply broad sonority restrictions on structures that are unattested in their language [34]–[44]. Moreover, sign languages, complete with both phonological and morphological patterns, emerge *de novo* in the human species [69]–[76]. Signed and spoken languages likewise share common developmental precursors [77] and brain mechanisms [78]–[86], and at least one of these mechanisms—the capacity to encode phonetic contrasts categorically—is present in all human infants [51], [52]. Our present conclusions converge with those past results to suggest that the design of the phonological system might be biologically determined by principles that are partly amodal [87].

## Methods

### Participants

Four groups of adult participants (N = 15 per group) took part in Experiments 1–4, respectively. Participants in Experiment 1 were Deaf individuals who were fluent in American Sign Language (ASL). Experiments 2–4 employed three groups of nonsigners who were English speakers and were not fluent in any sign language.

Deaf signer participants were from the greater Boston area. All were deaf adults who considered ASL to be their primary language, and all were well integrated into the Deaf community. Most (14/15) Deaf participants acquired a sign language before the age of five, and one learned it at the age of eight. Eleven participants first acquired ASL, one participant acquired Mexican Sign Language, and three acquired Signed Exact English (a sign language that is a hybrid of ASL signs and English syntax). Participants were paid $20 for their participation.

The three Nonsigner groups were hearing individuals, students at Northeastern University. They took part in this experiment in partial fulfillment of course credit. Participants were questioned on their command of sign languages, and none has reported any fluency. Two participants (in Experiment 4) reported knowledge of a single sign, and three reported knowledge of the ASL alphabet (one in Experiment 3 and two in Experiment 4). One additional participant who reported taking an ASL college course was excluded from Experiment 2, and replaced by another hearing person who did not have any knowledge of a sign language.

Participants in Experiments 1–4 were presented with the same materials and procedures—the experiments only differed in the feedback provided to participants in the practice session (no group received any feedback during the experimental session). Experiments 1 & 3 provided Deaf and English speaking (nonsigner) participants with feedback on their accuracy concerning syllable- and morpheme count, such that syllable- and morpheme count are determined by the number of movements and handshapes, respectively; Experiment 2 provided no feedback to participants (nonsigners), whereas in Experiment 4 provided reverse feedback, pairing syllables with handshape, and morphemes with movement.

This study was approved by the IRB at Northeastern University. Written informed consent was obtained from all participants.

### Materials

The materials were short video clips featuring novel ASL signs. All signs were phonotactically legal, but they did not correspond to any existing ASL signs. These signs were comprised of four types, generated by crossing the number of syllables (one vs. two) and morphemes (one vs. two).

Syllable structure was manipulated by varying the number of movements (one vs. two movements), whereas morpheme structure was defined by the number of handshapes (one vs. two handshapes)—an association that is clearly evident in the structure of ASL compounds. We chose to model the morphological structure of our materials after the structure of ASL compounds because its linear morphological organization mirrors the linear organization of syllables. It should be noted, however, that the morphological structure of ASL is of often multi-linear (or nonconcatenative)[12], [13], and it can be further realized by movement and location [11], [88]–[92]. Likewise, syllable structure has been associated with changes in handshape aperture (closed <-> open) or changes in the orientation of the wrist, which are also a type of movement. Our hypothesis does not state that syllables are *only* linked to path movement, but our stimulus items were constructed using only path movement for simplicity.

In as much as possible, monosyllabic and disyllabic signs were matched for handshape and location and contrasted by the number of movements (In some cases there was an additional transitional phonetic movement. For example, a movement away from one shoulder followed by a movement from the other shoulder has a transitional movement to move the hand across the body). Similarly, within each such quartet, monomorphemic and bimorphemic signs were matched for location and handshape, and contrasted by handshape configuration (monomorphemes had one group of active fingers; bimorphemes had two groups). The experimental materials consisted of 13 quartets of novel signs (see Table S1 in Supporting Information S1). Two additional quartets were included in the experiment, but they were removed from all analyses because they did not exhibit the intended number of movements and handshapes. The matching of these monosyllabic and disyllabic items for handshape, movement, location and palm orientation is described in Table S2 in Supporting Information S1.

These materials were submitted to two tasks, administered in separate blocks of trial. Participants were first instructed to count the number of syllables in these items, next they were asked to count the number of morphemes. Prior to their participation in the experiment with novel signs, participants took part in two additional blocks of trials, identical in design to the ones with novel trials, except that those blocks featured existing ASL signs (60 trials per block). The syllable count of novel signs was preceded by counting syllables in existing ASL signs. Likewise, morpheme count of novel signs was preceded by morpheme count of existing ASL signs. Because responses to existing signs might be based on the familiarity of Deaf participants with these particular items, they do not necessarily reflect productive linguistic principles—the main focus of our present research. For the sake of brevity, we do not report these findings here. However, the results with existing signs and novel signs were similar.

All participants were provided with detailed instructions followed by practice. The block trials of ASL signs was preceded by practice with 8 ASL signs, whereas the subsequent block of novel signs was preceded by practice with 8 novel signs. Like the experimental trials, the practice list comprised equal combinations of 2 syllables (one vs. two) ×2 morphemes (one vs. two). The structure of practice items (ASL signs and novel signs) is provided in Tables S4 and S5 in Supporting Information S1, respectively. Nonsigner participants were given the same blocks of sign-trials as the signers (practice and experimental sessions with both ASL signs and novel signs), but prior to the presentation of signs, they were given brief practice with English stimuli. Specifically, nonsigners were first given practice with 8 English words, followed by the block of existing ASL signs (first practice, then the experimental trials). Likewise, the subsequent block of trials with nonwords first presented practice with 8 English nonwords, followed by the block of novel ASL signs (first practice, and then the experimental trials). None of the experimental trials with novel ASL appeared in the practice session. Likewise, most of the real ASL signs presented in the experimental session did not overlap with the practice items (the only exception was the sign for WIFE, which was repeated in both sessions).

All stimuli consisted of video recordings of a female, native signer of ASL. The duration of the four types of signs is provided in Table S3 in Supporting Information S1. All stimuli were inspected by a linguist who is fluent in ASL (DB) to assure that the number of syllables and morphemes in these signs is as intended, and that those novels signs are phonotactically well-formed. Examples of novel signs can be found on http://www.youtube.com/playlist?list=PLBamIsRMHpt3cFJ_XDH78jEdwkZEVXWDy

### Instructions

Prior to the experiment, participants were presented with instructions, designed to explain the experimental task and clarify the terms “syllable” and “morpheme” for both the Deaf signers and the Nonsigner participants. The instructions for the Deaf signers were presented in ASL. They were videotaped, and produced by the same native signer who also generated the experimental materials. Nonsigner participants were read an English version of the same instructions. The ASL instructions were inspected for clarity and naturalness by a linguist who is fluent in ASL (DB).

#### Instructions syllable count

The instructions for the syllable-count task first explained that ASL signs comprise meaningless parts—either one such part or two. Participants were provided with examples of existing ASL signs that are either monosyllabic or disyllabic. They were informed that these signs might also comprise meaningful units, but they were asked to ignore those meaningful units for the purpose of this experiment, and only focus on meaningless parts. Participants were then given practice on the syllable count task with ASL signs. The main experiment with novel signs followed. Participants were told that the task remains the same, except that “the signs you will see now are new—they do not actually exist in American Sign Language, but we think they are possible signs”. They were next provided eight practice trials with novel ASL signs, followed by the experimental session. The video recordings of the ASL instructions are provided in http://www.youtube.com/playlist?list=PLBamIsRMHpt04Lcnq42sZ8×2ejf1Dzt1Q; and their translation back into English is given in Appendix S1 in Supporting Information S1.

Instructions for the Nonsigners were similar, except that participants were also given examples of “meaningless” vs. “meaningful” chunks in English words (e.g., “sport” has one chunk whereas “support” has two; “sports” has two pieces of meaning—the “sport” part and the plural part “-s”. Likewise, “supports” includes the base “support” and a plural “-s”). The full instructions for English speaking participants are presented in Appendix S2 in Supporting Information S1.

All participants were asked to base their response on the way the sign is produced in the video, and watch the video carefully. They were told to press the “1 key” if the signs have one chunk, and press “the 2 key” for signs with two chunks. After the instructions, participants were presented with a short practice with eight existing signs, and invited to ask questions. Participants were advised to “respond as fast and accurately as you can—don’t try to over-analyze; just go with your gut feeling”.

#### Instructions for morpheme count

The instructions for the morpheme count task were similar to the syllable count task, except that people were now asked to determine whether this word has one piece of meaning or two. They were informed that the signs might also contain meaningless parts and advised to ignore this fact and focus only on meaningful pieces. Note that, in all conditions, participants were only informed of the distinction between meaningful and meaningless chunks—they were never provided any explicit information on how this distinction is implemented in ASL (i.e., by the number of movements or handshapes). All participants were asked to respond as fast and accurately as they could “don’t try to over-analyze; just go with your gut feeling”.

### Procedure

Participants were seated in front of a computer. Each trial began with a fixation point (+) presented for 500 ms, followed by a short video clip. Participants responded by pressing the appropriate key (1 = one chunk; 2 = chunks). Participants had up to 5 seconds to respond from the onset of the video, and their response triggered the next trial. Participants were tested either individually, or in small groups of up to four participants.

Prior to the experimental trials, all participants were given practice with ASL signs, and Nonsigners also received practice with English stimuli.

During practice, Signers in Experiment 1 and Nonsigner participants in Experiments 3–4 were presented with feedback on their accuracy with ASL signs. In Experiments 1 and 3, correct syllable count responses were determined by the number of movements (one movement per syllable) whereas correct morpheme count responses were determined by the number of handshapes (one handshape per morpheme). In Experiment 4, feedback enforced the reverse correspondence. Thus, correct syllable count was determined by the number of handshapes (one handshape per syllable), whereas correct morpheme count was determined by the number of movements (one movement per morpheme).

When feedback was provided (i.e., in the practice sessions of Experiments 1, 3 & 4), correct responses triggered the message “correct”. Incorrect feedback messages that pointed out the different chunks/meaningful parts in the stimulus. To use an English example, an incorrect “one chunk” response to the word “blackboard” would trigger the message “Sorry, The word "blackboard" has 2 chunk(s): black – board. Press space bar to try again. “, followed by another presentation of the same sign. Thus, feedback messages clarified the segmentation of the sign, but they did not explain how segments are defined (i.e., by the movement/handshape of ASL signs). Nonsigner participants in Experiments 3–4 also received similar feedback on their practice with English words and nonwords, but here, the feedback was always consistent with the structure of English syllables and morphemes. No group received feedback during the experimental session.

## Supporting Information

Supporting Information S1 **Supporting tables and appendices. Table S1.** The structure of the novel ASL signs used in Experiments 1–4. **Table S2.** The matching of monosyllabic and disyllabic novel ASL signs for the handshape, location, palm orientation and movement. **Table S3.** The duration (in seconds) of the novel ASL signs in Experiments 1–4. **Table S4.** The existing ASL signs employed in the practice session. **Table S5.** The novel ASL signs employed in the practice session. **Appendix S1.** The instructions presented to ASL signers in Experiment 1 (translated back into English). **Appendix S2.** The instructions presented to English speakers.
(PDF)

## References

[1] Hockett CF (1960) The origin of speech. Sci Am 203: 89–96. 14402211

[2] Prince A, Smolensky P (2004) Optimality theory: Constraint interaction in generative grammar. (Originally published as a technical report in 1993.) Malden,MA : Blackwell Pub.

[3] McCarthy JJ, Prince A (1998) Prosodic morphology. In: Spencer A, Zeicky AM, editors. Handbook of Morphology.Oxford: Basil Blackwell. pp. 283–305.

[4] Fitch WT, Hauser MD, Chomsky N (2005) The evolution of the language faculty: clarifications and implications. Cognition 97: : 179–210; discussion 211-125. 10.1016/j.cognition.2005.02.005 16112662

[5] MacNeilage PF (2008) The origin of speech. New York:Oxford University Press .xi, 389 p.

[6] Pinker S, Jackendoff R (2005) The faculty of language: What's special about it? Cognition 95: 201–236. 15694646 10.1016/j.cognition.2004.08.004

[7] Clements GN (1990) The role of the sonority cycle in core syllabification. In: Kingston J, Beckman M, editors. Papers in laboratory phonology I: Between the grammar and physics of speech.Cambridge:Cambridge University Press. pp. 282–333.

[8] Steriade D (1988) Reduplication and syllable transfer in Sanskrit and elsewhere. Phonology 5: 37–155.

[9] Parker S (2002) Quantifying the Sonority Hierarchy [doctoral dissertation]. Amherst,MA:University of Massachusetts.

[10] Stokoe WC Jr (1960) Sign Language Structure: An Outline of the Visual Communication Systems of the American Deaf. Journal of Deaf Studies and Deaf Education 10: 3–37. 10.1093/deafed/eni001 15585746

[11] Klima ES, Bellugi U (1979) The signs of language. Cambridge, MA: Harvard University Press.

[12] Sandler W, Lillo-Martin DC (2006) Sign language and linguistic universals. Cambridge:Cambridge University Press . xxi, 547 p.

[13] Brentari D (1998) A prosodic model of sign language phonology. Cambridge,MA:MIT Press.xviii, 376 p.

[14] Perlmutter DM (1992) Sonority and syllable structure in American Sign Language. Linguistic Inquiry: 407–442.

[15] Corina DP (1990) Reassessing the role of sonority in syllable structure: Evidence from visual gestrual language. In: Ziolkowski M, Noske M, Deaton K, editors.Papers From the 26th Annual Regional Meeting of the Chicago Linguistic Society.Chicago:University of Chicago. pp. 33–43.

[16] Smolensky P (2006) Optimality in Phonology II: Harmonic completeness, local constraint conjunction, and feature domain markedness. In: Smolensky P, Legendre G, editors. The harmonic mind: From neural computation to Optimality-theoretic grammar.Cambridge,MA :MIT Press. pp. 27–160.

[17] Ohala JJ (1990) Alternatives to the Sonority Hierarchy for Explaining Segmental Sequential Constraints. Papers from the Regional Meetings, Chicago Linguistic Society 2: 319–338.

[18] Zec D (2007) The syllable. In: de Lacy P, editor. The Cambridge handbook of phonology.Cambridge:Cambridge University Press. pp. 161–194.

[19] de Lacy P (2006) Markedness: reduction and preservation in phonology. New York:Cambridge University Press. xviii, 447 p.

[20] Brentari D (1993) Establishing a sonority hierarhcy in American Sign Language: the use of simultaneous structure in phonology. Phonology 10: 281–306.

[21] Brentari D (1994) Prosodic constraints in American Sign Language. In: Bos H, Schermer T, editors.Sign Language Research. Hamburg: Signum Press. pp. 39–51.

[22] Jantunen T, Takkinen R (2010) Syllable structure in sign language phonology. In: Brentari D, editor.Sign languages.Cambridge:Cambridge University Press. pp. 312–331.

[23] Corina D, Sandler W (1993) On the Nature of Phonological Structure in Sign Language. Phonology 10: 165–207.

[24] Sandler W (2008) The syllable in sign language: considering the other natural language modality. In: Davis BL, Zajdó K, editors.The Syllable in Speech Production. New York:Lawrence Erlbaum Associates. pp. 379–408.

[25] Wilbur R (2012) Sign Syllables. In: van Oostendorp M, Ewen CJ, Hume E, Rice K, editors. The Blackwell Companion to Phonology.London: Blackwell Publishing. pp. 1309–1334.

[26] Brentari D (2006) Effects of language modality on word segmentation: An experimental study of phonological factors in a sign language. In: Goldstein L, Whalen D, Best C, editors.Papers in Laboratory Phonology VIII .Berlin:Mouton de Gruyter. pp. 155–164.

[27] Ashby J, Rayner K (2004) Representing syllable information during silent reading: Evidence from eye movements. Language and Cognitive Processes 19: 391–426.

[28] Carreiras M, Alvarez CJ, de Vega M (1993) Syllable frequency and visual word recognition in Spanish. Journal of Memory and Language 32: 766–780.

[29] Cholin J (2011) Do syllables exist? Psycholinguistic evidence of the retrieval of syllabic units in speech production. In: Cairns CE, Raimy E, editors.Handbook of the Syllable Brill. pp. 225–248.

[30] Coetzee A (2011) Syllables in speech perception—evidence from perceptual epenthesis In: Cairns C, Raimy E, editors. Handook of the Syllable. Leiden:E.J. Brill. pp.295–325.

[31] Conrad M, Carreiras M, Tamm S, Jacobs AM (2009) Syllables and bigrams: Orthographic redundancy and syllabic units affect visual word recognition at different processing levels. Journal of Experimental Psychology: Human Perception and Performance 35: 461–479. 19331501 10.1037/a0013480

[32] Treiman R, Fowler C, Gross J, Berch D, Weatherston S (1995) Syllable structure or word structure? Evidence for onset and rime units with disyllabic and trisyllabic stimuli. Journal of Memory and Language 34: 132–155.

[33] Bertoncini J, Mehler J (1981) Syllable as units in infant speech perception. Infant behavior and development 4: 247–260.

[34] Berent I, Steriade D, Lennertz T, Vaknin V (2007) What we know about what we have never heard: Evidence from perceptual illusions. Cognition 104: 591–630. 16934244 10.1016/j.cognition.2006.05.015

[35] Berent I, Lennertz T, Jun J, Moreno MA, Smolensky P (2008) Language universals in human brains. Proceedings of the National Academy of Sciences 105: 5321–5325. 10.1073/pnas.0801469105 PMC2291138 18391198

[36] Berent I, Lennertz T, Smolensky P, Vaknin-Nusbaum V (2009) Listeners' knowledge of phonological universals: Evidence from nasal clusters. Phonology 26: 75–108. 21874095 10.1017/S0952675709001729 PMC3160771

[37] Berent I, Balaban E, Lennertz T, Vaknin-Nusbaum V (2010) Phonological universals constrain the processing of nonspeech. Journal of Experimental Psychology: General 139: 418–435. 20677893 10.1037/a0020094 PMC3258023

[38] Berent I, Harder K, Lennertz T (2011) Phonological universals in early childhood: Evidence from sonority restrictions. Language Acquistion 18: 281–293. 10.1080/10489223.2011.580676 PMC3275087 22328807

[39] Berent I, Lennertz T, Balaban E (2012) Language universals and misidentification: A two way street. Language and Speech 55: 1–20. 10.1177/0023830911417804 PMC3481201 23094317

[40] Berent I, Lennertz T, Rosselli M (2012) Universal phonological restrictions and language-specific repairs: Evidence from Spanish. The Mental Lexicon 13: 275–305.

[41] Pertz DL, Bever TG (1975) Sensitivity to phonological universals in children and adolescents. Language 51: 149–162.

[42] Ohala DK (1999) The influence of sonority on children's cluster reductions. Journal of communication disorders 32: 397–421. 10560714 10.1016/s0021-9924(99)00018-0

[43] Berent I, Lennertz T (2010) Universal constraints on the sound structure of language: Phonological or acoustic? Journal of Experimental Psychology: Human Perception & Performance 36: 212–223. 20121305 10.1037/a0017638

[44] Berent I, Lennertz T, Smolensky P (2011) Markedness and misperception: It's a two-way street. In: Cairns CE, Raimy E, editors.Handbook of the Syllable Leiden,The Netherlands: Brill. pp. 373–394.

[45] Lane H, Boyes-Braem P, Bellugi U (1976) Preliminaries to a distinctive feature analysis of handshapes in American sign language. Cognitive Psychology 8: 263–289.

[46] Hildebrandt U, Corina D (2002) Phonological Similarity in American Sign Language. Language and Cognitive Processes 17: 593–612.

[47] Emmorey K, McCullough S, Brentari D (2003) Categorical perception in American Sign Language. Language and Cognitive Processes 18: 21–45.

[48] Baker SA, Idsardi WJ, Golinkoff RM, Petitto I-A (2005) The perception of handshapes in American sign language. Memory & Cognition 33: 887–904. 16383176 10.3758/bf03193083 PMC2730958

[49] Newport E (1982) Task specificity in language learning? Evidence from speech perception and American Sign Language. In: Wanner E, Gleitman L, editors.Language acquisition: The state of the art. Cambridge: Cambridge University Press. pp. 450–486.

[50] Best CT, Mathur G, Miranda KA, Lillo-Martin D (2010) Effects of sign language experience on categorical perception of dynamic ASL pseudosigns. Attention, Perception, & Psychophysics 72: 747–762. 10.3758/APP.72.3.747 20348580

[51] Baker SA, Michnick Golinkoff R, Petitto L-A (2006) New Insights Into Old Puzzles From Infants' Categorical Discrimination of Soundless Phonetic Units. Language Learning and Development 2: 147–162. 19823599 10.1207/s15473341lld0203_1 PMC2759762

[52] Palmer SB, Fais L, Golinkoff RM, Werker JF (2012) Perceptual narrowing of linguistic sign occurs in the 1st year of life. Child Development 83: 543–553. 22277043 10.1111/j.1467-8624.2011.01715.x

[53] Orfanidou E, Adam R, Morgan G, McQueen JM (2010) Recognition of signed and spoken language: Different sensory inputs, the same segmentation procedure. Journal of Memory and Language 62: 272–283.

[54] Wilbur RB, Nolen SB (1986) The duration of syllables in American Sign Language. Language And Speech 29: 263–280. 3695760 10.1177/002383098602900306

[55] Wilbur RB, Petersen L (1997) Backwards signing and ASL syllable structure. Language And Speech 40: 63–90. 9230699 10.1177/002383099704000104

[56] Brentari D, González C, Seidl A, Wilbur R (2011) Sensitivity to visual prosodic cues in signers and nonsigners. Language And Speech 54: 49–72. 21524012 10.1177/0023830910388011

[57] Wilbur RB, Allen GD (1991) Perceptual Evidence against Internal Structure in American Sign Language Syllables. Language And Speech 34: 27–46. 1819681 10.1177/002383099103400102

[58] Pinker S (1991) Rules of language. Science 253: 530–535. 1857983 10.1126/science.1857983

[59] Pinker S (1997) Words and rules in the human brain. Nature. pp. 547–548. 10.1038/42347 9177332

[60] Berent I, Pinker S (2007) The dislike of regular plurals in compounds: Phonological familiarity or morphological constraint? The Mental Lexicon 2: 129–181.

[61] Berent I, Shimron J (1997) The representation of Hebrew words: Evidence from the Obligatory Contour Principle. Cognition 64: 39–72. 9342931 10.1016/s0010-0277(97)00016-4

[62] Berent I, Everett DL, Shimron J (2001) Do phonological representations specify variables? Evidence from the Obligatory Contour Principle. Cognitive Psychology 42: 1–60. 11161416 10.1006/cogp.2000.0742

[63] Berent I, Shimron J, Vaknin V (2001) Phonological constraints on reading: Evidence from the Obligatory Contour Principle. Journal of Memory and Language 44: 644–665.

[64] Berent I, Bibi U, Tzelgov J (2006) The autonomous computation of linguistic structure in reading: Evidence from the Stroop task. The Mental Lexicon 1: 201–230.

[65] Liddell S, Johnson R (1986) American Sign Language compound formation processes, lexicalization and phonological remnants. Natural Language and Linguistic Theory 4: 445–513.

[66] Jaeger TF (2008) Categorical data analysis: Away from ANOVAs (transformation or not) and towards logit mixed models. Journal of Memory and Language 59: 434–446. 19884961 10.1016/j.jml.2007.11.007 PMC2613284

[67] Berent I, Pinker S, Tzelgov J, Bibi U, Goldfarb L (2005) Computation of semantic number from morphological information. Journal of Memory and Language 53: 342–358.

[68] Hayes B, Kirchner RM, Steriade D, editors (2004) Phonetically based phonology.Cambridge: Cambridge University Press.

[69] Sandler W, Aronoff M, Meir I, Padden C (2011) The gradual emergence of phonological form in a new language. Natural Language and Linguistic Theory 29: 505–543. 10.1007/s11049-011-9128-2 PMC3250231 22223927

[70] Brentari D, Coppola M, Mazzoni L, Goldin-Meadow S (2012) When does a system become phonological? Handshape production in gestures, signers and homesigners. Natural Language & Linguistic Theory 30.. 10.1007/s11049-011-9145-1 PMC3665423 23723534

[71] Goldin-Meadow S, Mylander C (1983) Gestural communication in deaf children: noneffect of parental input on language development. Science 221: 372–374. 6867713 10.1126/science.6867713

[72] Goldin-Meadow S, Mylander C (1998) Spontaneous sign systems created by deaf children in two cultures. Nature 391: 279–281. 9440690 10.1038/34646

[73] Goldin-Meadow S (2003) The Resilience of Language: What gesture creation in deaf children can tell us about how all children learn language. New York:Psychology Press.

[74] Senghas A, Kita S, Ozyurek A (2004) Children creating core properties of language: evidence from an emerging sign language in Nicaragua. Science 305: 1779–1782. 15375269 10.1126/science.1100199

[75] Sandler W, Meir I, Padden C, Aronoff M (2005) The emergence of grammar: systematic structure in a new language. Proc Natl Acad Sci U S A 102: 2661–2665. 15699343 10.1073/pnas.0405448102 PMC548320

[76] Singleton JL, Newport EL (2004) When learners surpass their models: the acquisition of American Sign Language from inconsistent input. Cognit Psychol 49: 370–407. 15342259 10.1016/j.cogpsych.2004.05.001

[77] Petitto LA, Holowka S, Sergio LE, Ostry D (2001) Language rhythms in baby hand movements. Nature 413: 35–36. 11544514 10.1038/35092613

[78] Petitto LA, Zatorre RJ, Gauna K, Nikelski EJ, Dostie D, et al (2000) Speech-like cerebral activity in profoundly deaf people processing signed languages: implications for the neural basis of human language. Proc Natl Acad Sci U S A 97: 13961–13966. 11106400 10.1073/pnas.97.25.13961 PMC17683

[79] Nishimura H, Hashikawa K, Doi K, Iwaki T, Watanabe Y, et al (1999) Sign language 'heard' in the auditory cortex. Nature 397: 116. 9923672 10.1038/16376

[80] Newman AJ, Supalla T, Hauser PC, Newport EL, Bavelier D (2010) Prosodic and narrative processing in American Sign Language: an fMRI study. Neuroimage 52: 669–676. 20347996 10.1016/j.neuroimage.2010.03.055 PMC2908987

[81] MacSweeney M, Waters D, Brammer MJ, Woll B, Goswami U (2008) Phonological processing in deaf signers and the impact of age of first language acquisition. Neuroimage 40: 1369–1379. 18282770 10.1016/j.neuroimage.2007.12.047 PMC2278232

[82] MacSweeney M, Campbell R, Woll B, Giampietro V, David AS, et al (2004) Dissociating linguistic and nonlinguistic gestural communication in the brain. Neuroimage 22: 1605–1618. 15275917 10.1016/j.neuroimage.2004.03.015

[83] Emmorey K, Mehta S, Grabowski TJ (2007) The neural correlates of sign versus word production. Neuroimage 36: 202–208. 17407824 10.1016/j.neuroimage.2007.02.040 PMC1987366

[84] Emmorey K (2002) Language, cognition, and the brain: Insights from sign language research.Mahwah,NJ :Lawrence Erlbaum Associates Publishers.

[85] Corina DP, San Jose-Robertson L, Guillemin A, High J, Braun AR (2003) Language lateralization in a bimanual language. J Cogn Neurosci 15: 718–730. 12965045 10.1162/089892903322307438

[86] Corina DP, McBurney SL, Dodrill C, Hinshaw K, Brinkley J, et al (1999) Functional roles of Broca's area and SMG: evidence from cortical stimulation mapping in a deaf signer. Neuroimage 10: 570–581. 10547334 10.1006/nimg.1999.0499

[87] Berent I (2013) The phonological mind. Cambridge: Cambridge University Press.

[88] Supalla T (1982) Structure and Acquisition of verbs of motion and location in American Sign Language [PhD dissertation]: University of California, San Diego.

[89] Aronoff M, Meir I, Sandler W (2005) The Paradox of Sign Language Morphology. Language and Speech 81: 301–344. 10.1353/lan.2005.0043 PMC3250214 22223926

[90] Brentari D, editor (2010) Sign Languages: A Cambridge Language Survey.Cambridge:Cambridge University Press.

[91] Mathur G, Rathmann C (2011) Two types of nonconcatenative morphology in sign languages. In: Mathu G, Napoli DJ, editors. Deaf Around the World: The Impact of Language.Oxford:Oxford University Press. pp. 54–82.

[92] Pfau R, Steinbach M, Woll B, editors (2012) Sign Language. An International Handbook (HSK–Handbooks of Linguistics and Communication Science).Berlin: Mouton de Gruyter.

