# Correction: Amodal Aspects of Linguistic Design

**DOI:** 10.1371/annotation/935f97a6-67f9-4331-a998-f94a62826194

**Published:** 2013-04-05

**Authors:** Iris Berent, Amanda Dupuis, Diane Brentari

The Editor's affiliation is incorrect. The correct affiliation for editor Steven Pinker is:

Harvard University, United States of America

