# Supplementary material for: Amodal Aspects of Linguistic Design
Source: PLoS One. 2013 Apr 3;8(4):e60617. doi: 10.1371/journal.pone.0060617 (PMC3616023; doi:10.1371/journal.pone.0060617)
Supplement: Supporting Information S1 — Supporting tables and appendices. Table S1. The structure of the novel ASL signs used in Experiments 1–4. Table S2. The matching of monosyllabic and disyllabic novel ASL signs for the handshape, location, palm orientation and movement. Table S3. The duration (in seconds) of the novel ASL signs in Experiments 1–4. Table S4. The existing ASL signs employed in the practice session. Table S5. The novel ASL signs employed in the practice session. Appendix S1. The instructions presented to ASL signers in Experiment 1 (translated back into English). Appendix S2. The instructions presented to English speakers. (PDF) [file pone.0060617.s001.pdf]

Amodal aspects of linguistic design

Supporting Information

Iris Berent[1], Amanda Dupuis[1], Diane Brentari[2]*

*To whom correspondence should be addressed

[1] Department of Psychology, Northeastern University, Boston, MA, USA
[2] Department of Linguistics, University of Chicago, Chicago, IL, USA

Address for correspondence:
Iris Berent
Department of Psychology
Northeastern University
125 Nightingale
360 Huntington Ave
Boston MA 02115
Phone (617) 373 4033
i.berent@neu.edu

Table S1. The structure of the novel ASL signs used in Experiments 1-4.

Quartet	Sign		Handshape		Location	Palm-orientation		Movement
	**Morphemes**	**Syllables**	**Dominant**	**Non-dominant**		**Dominant**	**Non-dominant**	
1	1	1	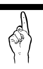		contralateral shoulder	down		straight path |->
	1	2	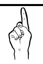		contralateral shoulder, ipsilateral shoulder	down		straight path |->, straight path |->
	2	1	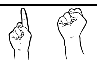		contralateral shoulder, head	down, out		straight path |->|
	2	2	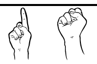		contralateral shoulder, head, neutral	down, out		straight path |-> , straight path |->
2	1	1	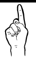	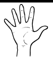	neutral	down	dominant	straight path ->
	1	2	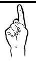	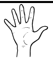	neutral	down	dominant	straight path repeat (2) ->, ->
	2	1	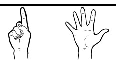	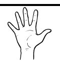	neutral, head	down, non-dominant	dominant	straight path |->|
	2	2	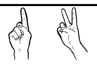	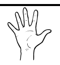	neutral, head	down, non-dominant	dominant	straight path,|-> straight path ->|
3	1	1	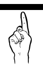	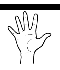	H2, neutral	dominant	non- minant	straight th |->
	1	2	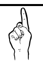	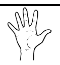	H2, neutral	dominant	non-dominant	straight path|-> straight path ->
	2	1	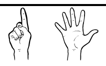	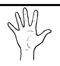	H2, chest	dominant	non-dominant	straight path |->|
	2	2	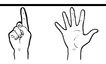	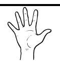	H2, neutral, chest	dominant	non-dominant	straight path |-> straight path ->|

4	1	1	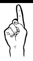		chest, neutral	down		arc |->
	1	2	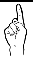		chest, neutral	down		arc |-> straight path ->
	2	1	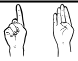		chest, nose	down		straight path |->|
	2	2	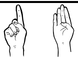		chest, neutral, nose	down		arc |-> straight path ->|
5	1	1	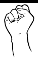		ear	out		straight path |->
	1	2	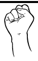		ear	out		straight path -> straight path -> (cross)
	2	1	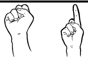	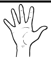	ear, H2	out, down	down	straight path |->|
	2	2	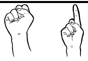	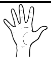	ear, neutral, H2	out, down	down	straight path |-> straight path ->|
6	1	1	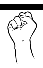		contralateral chest	down		straight path ->
	1	2	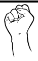		contralateral chest	down		straight path -> straight path -> (cross)
	2	1	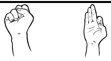		contralateral chest, ipsilateral head	down, out		straight path |->|
	2	2	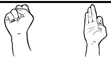		contralateral chest, ipsilateral head, neutral	down, out		straight path |->| straight path |->
7	1	1	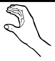		across face	non-dominant		arc ->
	1	2	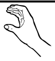		face	non-dominant		straight path -> straight

								path -> (cross)
	2	1	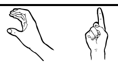		face, chest	non-dominant, down		straight path |->|
	2	2	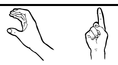		face, chest	non-dominant, down		arc -> straight path ->|
8	1	1	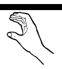		ipsilateral face	non-dominant		straight path ->
	1	2	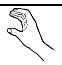		chin, neutral	non-dominant		straight path |-> straight path ->
	2	1	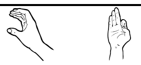		ipsilateral chin, contralateral chest	non-dominant		straight path |->|
	2	2	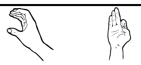		head, chin, chest	non-dominant		straight path -> straight path ->|
9	1	1	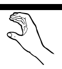		chest	non-dominant		tap ->|
	1	2	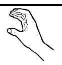		chest	non-dominant		circle -> tap ->|
	2	1	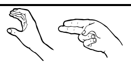	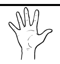	chest, H2	non-dominant	down	straight path |->|
	2	2	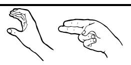	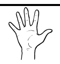	chest, H2	non-dominant	down	tap ->| straight path |->|
10	1	1	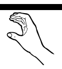	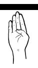	H2, neutral	non-dominant	dominant	straight path |->
	1	2	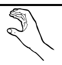	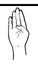	Neutral, H2	non-dominant	dominant	circle -> tap ->|
	2	1	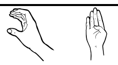	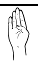	H2, face	non-dominant	dominant	straight path |->|
	2	2	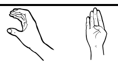	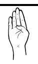	H2, neutral, face	non-dominant	dominant	arc |-> straight path ->|

11	1	1	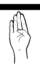		ipsilateral face	out		straight path ->
	1	2	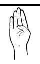		ipsilateral face	out		straight path -> straight path -> (cross)
	2	1	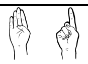	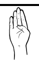	ipsilateral face, H2	out, in		straight path |->|
	2	2	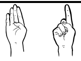	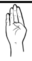	ipsilateral face, H2	out, in		straight path -> straight path ->|
12	1	1	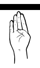	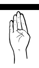	H2, neutral	in	dominant	arc ->
	1	2	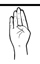	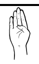	Neutral, H2	in	dominant	straight path |->| straight path |->
	2	1	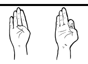	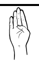	H2, contralateral chest	down, non-dominant	dominant	straight path |->|
	2	2	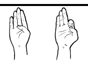	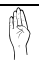	H2, contralateral chest	down, non-dominant	dominant	arc -> straight path ->|
13	1	1	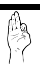		neutral, head	in		straight path ->|
	1	2	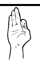		head, neutral	non-dominant		straight path |-> straight path |->
	2	1	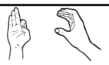	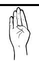	head, H2	non-dominant	down	straight path |->|
	2	2	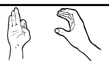	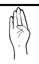	neutral, head, H2	non-dominant	down	straight path ->| straight path ->|

Note: All features are noted in the order in which they appear. "H2" refers to the nondominant, neutral hand. All palm-orientations are given from the perspective of the signer. Palm-orientations listed as “dominant” or “non-dominant” refer to a palm-orientation facing the dominant or non-dominant sides of the signing space, such that if a signer is right-handed “dominant” refers to “right.” |-> refers to movements directed away from the place of articulation; ->| refers to movements directed toward the place of articulation; |->| refers to movements directed away from the first place of articulation towards the second place of articulation; -> refers to movements that occur at the place of articulation and neither move away from or towards it.

Table S2. The matching of monosyllabic and disyllabic novel ASL signs for handshape, location, palm orientation and movement.

	**Monomorphemic**	**Bimorphemic**
**Handshape**		
Dominant	13/13	12/13
Neutral	4/4	7/7
**Location**	12/13	13/13
**Palm Orientation**		
Dominant	12/13	12/13
Neutral	4/4	7/7
**Movement**	10/13	13/13
**Total features matched**	54/60	63/66

Table S3. The duration (in seconds) of the novel ASL signs in Experiments 1-4.

	**Mean**	**SD**
One morpheme-one syllable	1.66	0.20
One morpheme-two syllables	2.10	0.37
Two morphemes-one syllable	1.73	0.22
Two morphemes-two syllables	2.05	0.21

<u>Table S4.</u> The existing ASL signs employed in the practice session.

Practice Quartet	Sign			Handshape		Location	Palm-orientation	Movement
	Gloss	Morphemes	Syllables	Dominant	Nondominant			
1	MANY	1	1	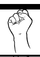 opens to 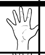	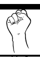 opens to 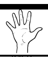	neutral	up,	opening
	EXPLOSION	1	2	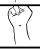 opens to 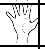	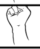 opens to 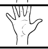	neutral	down, up	path, opening
	TRUST	2	1	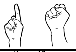	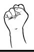	head, H2	in	straight path
	LIBRARIAN	2	2	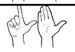	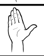	neutral, neutr	out, left	circle, straight path
2	MOTHER	1	1	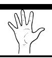		chin	left	tap
	FARM	1	2	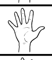		contralateral	left	tap, tap
	WIFE	2	1	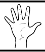	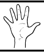	chin, H2	left	straight path
	PARENTS	2	2	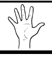		chin, forehead	left	tap, tap

Table S5. The novel ASL signs employed in the practice session.

Practice Quartet	Sign		Handshape		Location	Palm-orientation	Movement
	Morphemes	Syllables	Dominant	Nondominant			
1	1	1	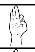		chest, neutral	out	arc
	1	2	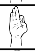		chest, neutral, neutral	down, out	arc, straight path
	2	1	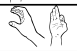	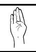	H2, chest	out, neutral - down	straight path
	2	2	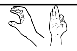	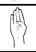	neutral, H2, chest	out, neutral - down	straight path, straight path
2	1	1	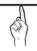	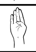	H2	down, neutral - down	straight path (wrist flick)
	1	2	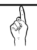	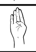	H2	down, neutral - down	straight path (wrist flick) repeat (2)
	2	1	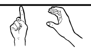	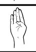	H2, head	down, left, neutral - down	arc
	2	2	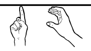	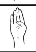	H2, neutral, head	down, left, neutral - down	straight path (wrist flick), arc

Appendix SI. The instructions presented to ASL signers in Experiment 1 (translated back into English).

Part 1 – Real word syllables. Video Instuct1.m4v

HELLO, WELCOME EXPERIMENT. NOW DO-DO? LIST$_{\text{two}}$ INDEX$_{\text{back and forth}}$. EXPERIMENT FOCUS WHAT? TOPIC, PARTS, HOW-MANY SEE.

KNOW-THAT ASL SIGN THEMSELVES PARTS HAVE. FEW SIGNS PARTS HAVE ONE. OTHER SIGNS, PARTS HAVE TWO.

ONE PART EXAMPLE. SIGN 'Y-O-U,' 'YOU.' HOW-MANY PARTS SEE? ONE. 'YOU.' TWO PART EXAMPLE SIGN 'H-O-S-P-I-T-A-L,' SIGN 'HOSPITAL.' HOW-MANY PARTS SEE? TWO. (first part of HOSPITAL) FIRST PART. (second part of HOSPITAL) SECOND PART. UNDERSTAND (first part of HOSPITAL) MEANING NONE, (second part of HOSPITAL) MEANING NONE. BUT ALTOGETHER SIGN 'HOSPITAL' MEANING 'H-O-S-P-I-T-A-L.'

UNDERSTAND, WARNING. NOW FOCUS MEANING$_{\text{negative}}$. MEANING IMPORTANT$_{\text{negative}}$. FOCUS WHAT? PARTS, HOW-MANY SEE. MEANING INSIDE, HOW MANY PARTS SEE SAME? NEGATE. DIFFERENT.

EXAMPLE SIGN ONE PART UNDERSTAND-CONDITIONAL TWO MEANING INSIDE. SIGN 'W-I-F-E.' 'WIFE' PARTS HOW-MANY HAVE? ONE. (PAUSE) WIFE. MEANING INSIDE HOW-MANY HAVE? TWO. THAT TWO SIGNS COMBINE BECOME ONE. SIGN 'WOMAN,' SIGN 'MARRY' COMBINE BECOME 'WIFE.' PARTS HOW-MANY SEE? ONE. MEANING INSIDE HOW-MANY? TWO. INDEXright INDEXleft SAME NEGATE DIFFERENT.

TWO PART EXAMPLE SIGN 'D-E-C-I-D-E' 'DECIDE.' PARTS HOW-MANY SEE? TWO. (PAUSE) DE-CIDE. MEANING INSIDE HOW-MANY? TWO. THAT TWO SIGNS COMBINE BECOME ONE. SIGN 'THINK,' SIGN 'ABSOLUTE' COMBINE BECOME 'DECIDE.' PARTS HOW-MANY SEE? TWO. MEANING INSIDE HOW-MANY? TWO. INDEXright, INDEXleft SAME (this is possible face).

REMEMBER EMPHASIZE MEANING IMPORTANT$_{\text{negative}}$ FOCUS WHAT? PARTS HOW-MANY SEE.

SUPPOSE SIGN SEE ONE PART HAVE, PRESS ONE. SUPPOSE SIGN SEE TWO PARTS HAVE, PRESS TWO.

WARNING, KNOW-THAT SOMETIMES YOUR PERSONAL SIGN, VIDEO MATCH-UP$_{\text{negative}}$. FINE. ACCEPT. BUT YOUR DECISION PRESS-ONE, PRESS-TWO DEPENDS WHAT? VIDEO. BE CAREFUL, WATCH.

OKAY, READY?

Hello, welcome to the experiment. This experiment will have two portions. The focus of the experiment will be on recognizable parts.

You may know that ASL signs contain recognizable parts. Some signs have one recognizable part, whereas other signs contain two.

An example of a sign with one part is the sign YOU, as shown by the signer. An example of a sign with two parts is the sign HOSPITAL, as shown by the signer. The first part of the sign has no meaning, nor does the second part. However, those two parts put together make up the sign HOSPITAL.

It is important to remember in this experiment to focus on visible parts, not meaning. For our purposes, meaning is inconsequential.

For example, there are signs that exist that are composed of two meanings, but only have one visible part. The sign WIFE is a prime example of this. WIFE is made up of the sign WOMAN combined with the sign MARRY. However, although there are two units of meaning within WOMAN, the sign WOMAN has only one visible part, as shown by the signer.

In contrast, there are also signs that have two units of meaning and two visible parts. An example of this would be the sign DECIDE. DECIDE is made up of the sign THINK combined with the sign ABSOLUTE. It also has two visible parts.

Thus, it is important to disregard meaning and focus on recognizable parts for the purposes of this experiment.

If you are shown a sign with one recognizable part, please press one. If you are shown a sign with two recognizable parts, please press two.

It is important to remember that although you may sign certain words differently than the signer in the video, your decisions should be based on the way the signer is producing the stimulus. That having been said, please watch the video closely.

Part 2 – Non word syllables. Video Instruct2.m4v

NOW LIST$_{\text{two}}$ INDEX$_{\text{second on list}}$, DO-DO? FOCUS SAME$_{\text{list}}$. FOCUS PARTS HOW-MANY SEE. INDEX$_{\text{second on list}}$ DIFFERENT WHY? SIGNS THEMSELVES VIDEO SHOW$_{\text{you}}$ TRUE$_{\text{negative}}$. SIGNS THEMSELVES MADE-UP. DOESN'T-MATTER, FOCUS PARTS HOW-MANY SEE. SUPPOSE SIGN CREATIVE SEE ONE PART PRESS ONE. IF SEE SIGN CREATIVE, TWO PARTS? PRESS TWO.

In the second portion of this experiment the task will be the same as in the first. The only difference is that the signs shown to you in the video will not be real ASL signs. Instead, they are signs carefully crafted to look similar to that which appear in ASL. This is inconsequential; your task is the same. If you see a sign with one part, please press one. If you see a sign with two parts, please press two.

Part 3 – Real word morphemes. Video Instruct3.m4v

HELLO WELCOME EXPERIMENT. NOW DO-DO LIST$_{\text{two}}$ INDEX$_{\text{list}}$. EXPERIMENT FOCUS WHAT? MEANING INSIDE HOW-MANY HAVE. KNOW-THAT ASL SIGNS SOMETIMES SIMPLE. OTHER SIGNS THEMSELVES TWO SIGNS COMBINE BECOME ONE.

SIMPLE EXAMPLE SIGN 'Y-O-U' 'YOU' SIMPLE. TWO SIGN COMBINE BECOME ONE EXAMPLE SIGN 'D-E-C-I-D-E' 'DECIDE.' THAT TWO SIGNS COMBINE BECOME ONE. SIGN 'THINK' SIGN 'ABSOLUTELY' COMBINE BECOME 'DECIDE.'

UNDERSTAND WARNING. KNOW-THAT ASL SIGN THEMSELVES HAVE PARTS SEE CAN. BUT DOESN'T-MATTER. FOCUS WHAT? MEANING HOW-MANY INSIDE. MEANING INSIDE, HOW MANY PARTS SEE SAME? NEGATE. DIFFERENT.

EXAMPLE SIGN TWO MEANING INSIDE UNDERSTAND-CONDITIONAL PARTS SEE HOW-MANY? ONE. SIGN 'W-I-F-E.' 'WIFE' MEANING INSIDE HOW-MANY HAVE? TWO. THAT TWO SIGNS COMBINE BECOME ONE. SIGN 'WOMAN,' SIGN 'MARRY' COMBINE BECOME 'WIFE.' BUT PARTS HOW-MANY SEE? ONE. (PAUSE) WIFE. MEANING INSIDE HOW-MANY? TWO. PARTS SEE HOW-MANY? ONE. INDEXright INDEXleft SAME NEGATE DIFFERENT.

EXAMPLE SIGN ONE MEANING INSIDE UNDERSTAND-CONDITIONAL PARTS SEE HOW-MANY? TWO. SIGN 'H-O-S-P-I-T-A-L' 'HOSPITAL.' MEANING INSIDE HOW-MANY? ONE. TWO SIGNS COMBINE BECOME ONE$_{negative}$. (first part of HOSPITAL) SIGN NOT. (second part of HOSPITAL) SIGN NOT. SIGN 'H-O-S-P-I-T-A-L' 'HOSPITAL' SIMPLE. TWO SIGNS COMBINE BECOME ONE$_{negative}$. BUT UNDERSTAND, PARTS SEE HOW-MANY? TWO. FIRST PART (first part of HOSPITAL), SECOND PART (second part of HOSPITAL) *EMPHASIZED. MEANING INSIDE HOW-MANY? ONE. PARTS SEE HOW-MANY? TWO. INDEXright INDEXleft SAME NEGATE DIFFERENT.

IF SEE SIGN SIMPLE, PRESS ONE. IF SEE SIGN TWO SIGNS COMBINE BECOME ONE, PRESS TWO.

WARNING. LET-YOU-KNOW. KNOW-THAT SOMETIMES YOUR PERSONAL SIGN, VIDEO SIGN MATCH-UP$_{negative}$. FINE. ACCEPT. BUT YOUR DECISION PRESS WHICH DEPENDS WHAT? VIDEO. TAKE-CARE, WATCH.

OKAY? READY?

Hello, welcome to the experiment. This experiment will have two portions. The focus of the experiment will be on units of meaning found within a sign.

As you may know, ASL signs contain units of meaning. Some signs only contain one unit of meaning and are therefore labeled as "simple." Other signs contain two units of meaning; they are two signs that have combined to become one sign and thus labeled "complex."

An example of a simple sign is the sign YOU, as shown by the signer. YOU only contains one unit of meaning and is therefore simple. An example of a complex sign is DECIDE, as shown by the signer. The sign DECIDE is made up of two signs, THINK and ABSOLUTE combined.

It is important to remember that in this experiment we are focusing on units of meaning rather than visible parts. Take for example the sign WIFE. Although WIFE only contains one visible part, it has two units of meaning. WIFE is a combination of the signs WOMAN and MARRY.

There are also signs that contain two visible parts and only one unit of meaning. An example of this is the sign HOSPITAL. While HOSPITAL has two visible parts, it would still be labeled as simple because it only contains one unit of meaning. Thus, for our purposes, it is important to ignore visible parts and focus only on units of meaning.

If you see a sign that is simple, please press one. If you see a sign that appears to be complex, please press two.

It is also important to remember that although you may sign certain words differently than the signer in the video, your decisions should be based on the way the signer is producing the stimulus. That having been said, please watch the video closely.

Part 4 – Non word morphemes. Video Instruct4.m4v

NOW DO-DO LIST$_{\text{two}}$ INDEX$_{\text{second on list}}$. SAMElist. FOCUS WHAT? MEANING INSIDE HOW-MANY. LIST$_{\text{two}}$ INDEX$_{\text{first, second}}$ DIFFERENT HOW? INDEX$_{\text{second on list}}$ SHOWyou SIGNS THEMSELVES REAL$_{\text{negative}}$. SIGNS THEMSELVES MADE-UP. LOOKS-LIKE SIMPLE OR LOOKS-LIKE TWO SIGNS COMBINE BECOME ONE MADE-UP SIGN.

IF SEE SIMPLE, PRESS ONE. IF SEE SIGN ITSELF LOOKS-LIKE TWO SIGN COMBINE BECOME ONE, PRESS TWO.

In the second portion of this experiment the task will be the same as in the first. The only difference is that the signs shown to you in the video will not be real ASL signs. Instead, they are signs carefully crafted to look similar to that which appear in ASL. This is inconsequential; your task is the same. If you see a sign that appears to be simple, please press one. If you see a sign that appears to be complex, please press two.

Appendix S2: The instructions presented to English speakers.

In this experiment, we ask you to judge some signs in American Sign Language. There are several different types of signs in American Sign Language, and your job will be to determine which type you see. The experiment as a whole has two large sections, each with multiple parts.

Section 1: syllable count condition

Some signs have one chunk whereas others have two. To use an example from English: The word "sport" has one chunk, while "support" has two. Please note that those chunks have no specific meaning, but we still perceive that "sport" has one chunk whereas "support" has two. Likewise, signs in American Sign Language may have either one chunk or two. We would like to find out whether English speakers can perceive this.

In addition to having different numbers of chunks, words can vary in how many pieces of meaning they have. Even in words with multiple pieces of meaning, you can still perceive different numbers of chunks. To use examples from English, the word "sports" has two pieces of meaning—the "sport" part and the plural part "-s". Likewise, "supports" includes the base "support" and a plural "-s". For your task here, however, the pieces of meaning are irrelevant. Only pay attention to how many chunks each word has: "Sports" is only one chunk, while the word "supports" has two meaningless chunks.

Real words

First you will practice the task with English words before moving on to American Sign Language. In each trial you will see one word. Your task is to determine whether this word has one chunk or two. If it has one chunk, press 1; if it has two, press 2. Please respond as fast and accurately as you can—don't try to over-analyze; just go with your gut feeling.

Any questions? Please press "z" to continue.

[participants receive the English practice]

Now for the American Sign Language practice. Like the English examples, these signs have either one chunk or two. We want to see whether you can guess how many are in each sign. Please watch the signer carefully and judge the signs exactly as they appear in the video.

As before, in each trial you will see one word. Each word is a real word in American Sign Language. If the sign has one chunk, press 1; if it has two, press 2. We know this is a hard task without knowing American Sign Language. Please try as best as you can.

Any questions? Please press "a" to continue.

[participants receive the ASL practice]

Good job on the practice! Now for the main part of the experiment. Your task is the same as in the practice: please determine if each sign has one chunk or two.

Any questions? Please press "q" to continue.

[participants receive the ASL word syllable condition]

Novel words

Thank you very much! Now we move to the second part of the experiment. This part is very similar to the previous one, except that the signs you will see now are new—they do not actually exist in American Sign Language, but we think they are possible signs. We ask you to determine whether they include one chunk or two. To explain what we mean, let's practice with similar examples from English.

In this practice, you will see nonsense English words. As before, do not base your answer on how many pieces of meaning the word might have. If the word has one chunk, press 1; if it has two, press 2. Please respond as fast and accurately as you can—don't over-analyze; just go with your gut feeling.

Any questions? Please press "e" to continue.

[participants receive the English non-word practice]
Now to practice with American Sign Language. Like the English examples you have just seen, these are nonsense signs in American Sign Language. Please determine if each sign has one chunk or two.
Any questions? Please press "d" to continue.
[participants receive the ASL non-word practice]
Now for the main part of the experiment. Your task is the same as in the practice: please determine if each sign has one chunk or two.
Any questions? Please press "c" to continue.
[participants receive the ASL non-word syllable condition]
You are now halfway done with the experiment; thank you for your hard work. The second half is similar, but asks you to change what aspect of the signs you are paying attention to.

Section 2: morpheme count condition
Some signs have one piece of meaning whereas others have two. To use an example from English: The word "sport" has one piece of meaning, while "sports" has two—the base "sport" and the plural "-s". Likewise, signs in American Sign Language may have either one piece of meaning or or two. We would like to find out whether English speakers can perceive this.
In addition to having different numbers of pieces of meaning, words can vary in how many chunks they have. Even in words with multiple chunks, you can still perceive different numbers of pieces of meaning. To use examples from English, the words "support" and "supports" both have two chunks. For your task here, however, the number of chunks is irrelevant. Only pay attention to how many pieces of meaning each word has: "Support" has only one piece of meaning, while "supports" has two.
Real words
First you will practice the task with English words before moving on to American Sign Language. In each trial you will see one word. Your task is to determine whether this word has one piece of meaning or two. If it has one piece of meaning, press 1; if it has two, press 2. Please respond as fast and accurately as you can—don't try to over-analyze; just go with your gut feeling.
Any questions? Please press "w" to continue.
Now for the American Sign Language practice. Like the English examples, these signs have either one piece of meaning or two. We want to see whether you can guess how many are in each sign. Please watch the signer carefully and judge the signs exactly as they appear in the video.
As before, in each trial you will see one word. Each word is a real word in American Sign Language. If the sign has one piece of meaning, press 1; if it has two, press 2. We know this is a hard task without knowing American Sign Language. Please try as best as you can.
Any questions? Please press "s" to continue.
[participants receive the ASL practice]
Good job on the practice! Now for the main part of the experiment. Your task is the same as in the practice: please determine if each sign has one piece of meaning or two.
Any questions? Please press "x" to continue.
[participants receive the ASL word morpheme condition]
Novel words
Thank you very much! Now we move to the second part of the experiment. This part is very similar to the previous one, except that the signs you will see now are new—they do not actually exist in American Sign Language, but we think they are possible signs. We ask you to determine whether they include one piece of meaning or two. To explain what we mean, let's practice with similar examples from English.
In this practice, you will see nonsense English words. As before, do not base your answer on how many chunks the word might have. If the word has one piece of meaning, press 1; if it has two, press 2. Please respond as fast and accurately as you can—don't over-analyze; just go with your gut feeling.
Any questions? Please press "v" to continue.
[participants receive the English non-word practice]
Now to practice with American Sign Language. Like the English examples you have just seen, these are nonsense signs in American Sign Language. Please determine if each sign has one piece of meaning or two.
Any questions? Please press "f" to continue.
[participants receive the ASL non-word practice]
Now for the main part of the experiment. Your task is the same as in the practice: please determine if each sign has one piece of meaning or two.
Any questions? Please press "r" to continue.

[participants receive the ASL non-word morpheme condition]
You are now done with the experiment; thank you for your hard work.
